# Mutual Relations between Substituent Effect, Hydrogen Bonding, and Aromaticity in Adenine-Uracil and Adenine-Adenine Base Pairs †

**DOI:** 10.3390/molecules25163688

**Published:** 2020-08-13

**Authors:** Paweł A. Wieczorkiewicz, Halina Szatylowicz, Tadeusz M. Krygowski

**Affiliations:** 1 Faculty of Chemistry, Warsaw University of Technology, Noakowskiego 3, 00-664 Warsaw, Poland; pawel.wieczorkiewicz.stud@pw.edu.pl; 2 Faculty of Chemistry, University of Warsaw, Pasteura 1, 02-093 Warsaw, Poland; tmkryg@chem.uw.edu.pl

**Keywords:** substituent effect, hydrogen bond, aromaticity, adenine

## Abstract

The electronic structure of substituted molecules is governed, to a significant extent, by the substituent effect (SE). In this paper, SEs in selected nucleic acid base pairs (Watson-Crick, Hoogsteen, adenine-adenine) are analyzed, with special emphasis on their influence on intramolecular interactions, aromaticity, and base pair hydrogen bonding. Quantum chemistry methods—DFT calculations, the natural bond orbital (NBO) approach, the Harmonic Oscillator Model of Aromaticity (HOMA) index, the charge of the substituent active region (cSAR) model, and the quantum theory of atoms in molecules (QTAIM)—are used to compare SEs acting on adenine moiety and H-bonds from various substitution positions. Comparisons of classical SEs in adenine with those observed in para- and meta-substituted benzenes allow for the better interpretation of the obtained results. Hydrogen bond stability and its other characteristics (e.g., covalency) can be significantly changed as a result of the SE, and its consequences are dependent on the substitution position. These changes allow us to investigate specific relations between H-bond parameters, leading to conclusions concerning the nature of hydrogen bonding in adenine dimers—e.g., H-bonds formed by five-membered ring nitrogen acceptor atoms have an inferior, less pronounced covalent nature as compared to those formed by six-membered ring nitrogen. The energies of individual H-bonds (obtained by the NBO method) are analyzed and compared to those predicted by the Espinosa-Molins-Lecomte (EML) model. Moreover, both SE and H-bonds can significantly affect the aromaticity of adenine rings; long-distance SEs on π-electron delocalization are also documented.

## 1. Introduction

Fundamental biological importance makes DNA and RNA base pairs important and therefore popular systems for quantum chemical calculations. With the development of quantum chemistry methods and computing power, modeling systems of large sizes became easily accessible [1]. Therefore, since the 1990s many papers on this topic have been published, including articles on hydrogen bonding [2,3], π-stacking interactions [4,5], tautomerization [6], benchmarks of various DFT methods [7], and dispersion models [8], leading to a better understanding of nucleic acid structure and the mechanisms of mutations [9].

Various quantum chemical methods were used to investigate intermolecular interactions in Watson-Crick [10], non-canonical Hoogsteen [11], and adenine-uracil RNA base pairs, as well as in mismatched adenine-adenine base pairs [4,5]. The substituent effects (SEs) on hydrogen bonding were most frequently studied in structurally modified Watson-Crick base pairs (adenine-thymine and guanine-cytosine) [12,13,14,15,16,17,18]. Recently, the influence of substituents on the electronic structure of the four most stable purine tautomers and their adenine analogues [19], as well as on the stability of adenine quartets [20] has been presented.

**Figure 1:**
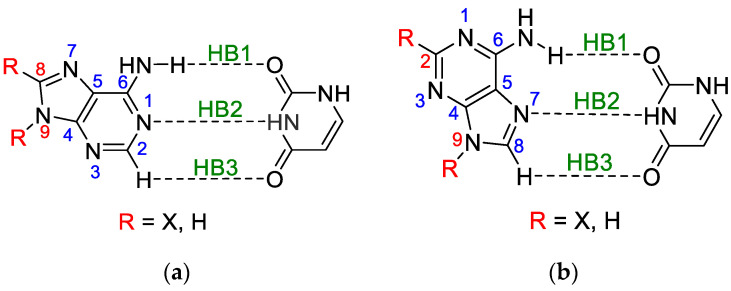
Adenine-uracil (**a**) Watson-Crick (abbreviated as WC) and (**b**) Hoogsteen (abbreviated as HG) base pairs with the common numbering of adenine atoms and adopted hydrogen bond numeration. Substitutable positions are marked in red color.

**Figure 2:**
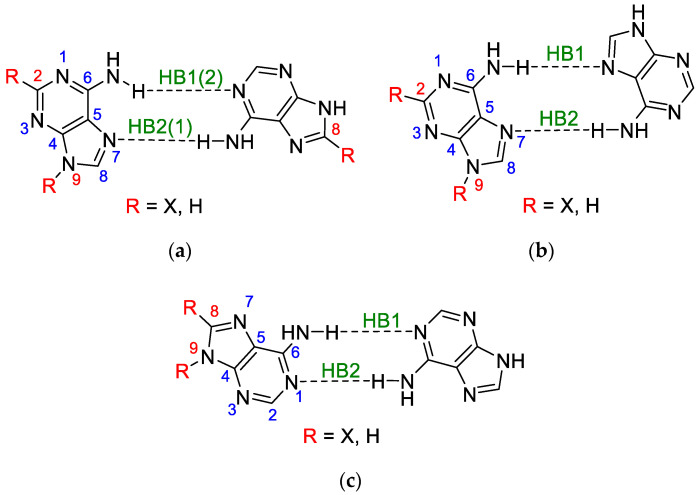
Adenine-adenine (**a**) AA2, (**b**) AA3, and (**c**) AA4 base pairs with the common numbering of adenine atoms and adopted hydrogen bond numeration (for AA2 C8-X dimer, numeration in brackets). Substitutable positions are marked in red color.

Adenine and uracil belong to five bases constituting DNA or RNA macromolecules that are fundamental to life processes [21]. Undoubtedly, the interactions between all of them, as well as the different types of external influences on their electronic structure, are of great importance for understanding their role in these processes. The influences of the electrophilic or nucleophilic agents belong to this type of interaction; they can cause significant changes in the electronic structure of the bases of DNA or RNA macromolecules. However, the interactions caused by cations or anions are temporary and difficult to systematically study. Their contact with the molecules in question, even if very short, is about 100 times longer than the time during which the electronic structure of the molecule is perturbed and open to some “non-typical” reactions with another reagent. Such situations can cause mutations in the attacked molecule and change its function as an inherent part of DNA or RNA macromolecules. To investigate this problem, instead of free-charged reagents, the attachment of electrophilic or nucleophilic groups (substituents) to a molecular moiety can be used to study their permanent effect on the electronic structure of the substituted molecule. Then, some analogies to a more complex situation can be subject to deeper consideration. The latter type of treatment will be presented in this paper, combined with the studies of mutual interactions of participants in adenine-uracil and adenine-adenine base pairs. In other words, the effect of intramolecular interactions (substituent effect) on individual intermolecular interactions (hydrogen bonds) in substituted base pairs is the subject of this paper. The studied adenine-uracil and adenine-adenine base pairs are shown in Figure 1 and Figure 2 (the separate structures of each dimer are shown in Figures S1–S4 in Supplementary Materials). The naming of the adenine dimers was adopted from the paper of Poater et al. [4] to allow the comparison of the results.

Several methods can be used to estimate the strength of individual hydrogen bonds in base pairs of nucleic acids: the rotational method [22], the compliance constants method [23], the Espinosa-Molins-Lecomte (EML) equation [24] application [25], the atom replacement method [26], the estimation of hydrogen bond energy based on electron density (calculated using the quantum theory of atoms in molecules, QTAIM) [27,28] at the bond critical point (BCP) [29], the application of natural bond orbitals (NBO) [30] method [31], and coordinates interaction approach, [32] as well as the delocalization index [33].

The most known substituent characteristics are the Hammett constants [34,35]. However, they can only be used to describe the classical substituent effect—how a substituent X affects the properties of a fixed group Y (the so-called “reaction site”) in a substituted system X-R-Y (R—transmitting moiety). The use of the cSAR (charge of the substituent active region) descriptor [36,37,38] allows us to study both classical and reverse substituent effects [39]; the latter describes how the electronic properties of substituents X depend on the properties of the moiety R-Y to which they are attached.

In the presented research, the most stable adenine tautomer, 9H, was chosen as a base for the further modification of the molecule. This tautomer has three substitutable hydrogen atoms at the C2, C8, and N9 positions. For each adenine-uracil and adenine-adenine base pair, substituent positions for further analysis were selected to avoid direct intermolecular interactions of a substituent, such as steric interactions of bulky substituents or the formation of a new hydrogen bond. For comparison, the effect of substituents at the C2, C8, or N9 positions in adenine on its physicochemical properties was also considered in detail. Selected substituents that differ in electronic properties (X = NO, NO_2_, Cl, F, H, Me, OH, NH_2_) were introduced into the adenine molecule in monomers and dimers.

The strength of the individual hydrogen bonds was characterized using the NBO approach [30,31], the topological parameters within the QTAIM approach [40], the delocalization index [33], and the H-bond lengths.

This work is mainly devoted to the influence of substituents on hydrogen bonding as well as on changes in the electronic structure of adenine, uracil, and their dimers (shown in Figure 1 and Figure 2). The following issues are considered in greater detail:

- The classical substituent effect—i.e., how do substituents in various positions of adenine affect its amino group and individual hydrogen bonds?
- How do substituents affect the π-electron delocalization in adenine rings?
- How does the electronic structure of the substituents, estimated by *c*SAR(X), depend on the position and kind of a moiety to which they are attached? This means the estimation of the so-called reverse substituent effect.
- How do these characteristics depend on the nature of substituents—i.e., their electron-donating or electron-attracting properties?
- How do these characteristics differ from those estimated for monomers?

## 2. Methods

For all studied systems, geometry optimizations without any symmetry constraints and electronic energy calculations were performed using the Gaussian 16 program [41]. Based on our previous research [42], the DFT-D method was used—namely, B97-D3 dispersion corrected density functional [43,44]—with Dunning’s [45] aug-cc-pVDZ basis set. The harmonic vibrational frequencies were calculated at the same level of theory to confirm that all the obtained structures correspond to the minima on the potential energy surface. No imaginary frequencies were found for the obtained series. In the case of asymmetric substituents (NO and OH), a lower energy rotamer was considered. NBO 6.0 software (Theoretical Chemistry Institute, University of Wisconsin, Madison, WI, USA) [46] was used for the NBO calculations.

The substituents were characterized using the *c*SAR descriptor. It allows a quantitative comparison of the electron-donating/withdrawing effects of different functional groups and correlates with many physicochemical properties [39]. For the X substituent, cSAR is defined as follows (Equation (1)):

(1) $$c\mathrm{SAR}\left(X\right)=Q_{X}+Q_{I},$$

where Q_X_ is the sum of the partial charges of the X group atoms, and Q_I_ is the partial charge of an atom to which a substituent is attached (ipso atom).

Partial charges calculated using the Hirshfeld, [47] Weinhold [30] (NBO), and the Voronoi Deformation Density (VDD) [48] methods were used to select the charge assessment for further investigation. Their values for derivatives of the WC base pair substituted in positions C8-X and N9-X were mutually correlated; the relations between *c*SAR(X) and *c*SAR(X)_Hir_ are shown in Figure S5 (Supplementary Materials). Considering both substitution positions (C8 and N9), it can be concluded that, qualitatively, only the VDD and Hirshfeld approaches are nearly equivalent for the estimation of *c*SAR(X) values. To be able to compare with the results of our previous research [19], only the Hirshfeld charges are used in further discussion and the superscript Hir is omitted.

The interaction energies between monomers A and B were obtained by the supermolecular method (Equations (2)–(4)) [49], using the counterpoise approach [50]:

(2) $$E_{\mathrm{SM}}=\;E_{\mathrm{AB}}-(E_{A}+E_{B})+E_{\mathrm{BSSE}}=E_{\mathrm{AB}}^{\mathrm{int}}+E_{\mathrm{AB}}^{\mathrm{def}},$$

(3) $$E_{\mathrm{AB}}^{\mathrm{int}}\;=\;E_{\mathrm{AB}}-\left(E_{A}^{\dim}+\;E_{B}^{\dim}\right),$$

(4) $$E_{\mathrm{AB}}^{\mathrm{def}}=E_{A}^{\dim}-\;E_{A}+E_{B}^{\dim}-\;E_{B},$$

where *E*_AB_ is the electronic energy of a dimer, whereas *E*_A_ and *E*_B_ are the electronic energies of monomers A and B, and *E*_BSSE_ is the basis set superposition error (BSSE) energy correction. *E*_AB_^int^ and *E*_AB_^def^ are the “pure” interaction and deformation energies in the AB dimer, respectively, while *E*_A_^dim^ and *E*_B_^dim^ are the energies of monomers A and B in the dimer geometry, respectively. For the studied systems, the *E*_BSSE_ values were 0.97–1.12 kcal/mol.

The energy of individual hydrogen bonds was calculated according to the NBO theory [30] as:

(5) $$E_{\mathrm{HB}}=E_{n\to \sigma *}-E_{n\to \sigma },$$

where *E*_n→σ*_ is the interaction energy between the nonbonding NBO orbital n (lone pair) of an H-bond acceptor atom and an antibonding orbital σ* of an H-D bond (where D is a hydrogen bond donor atom), calculated by the NBO 6.0 program from the second-order perturbative analysis of the Fock matrix on an NBO basis. *E*_n→σ_ is the steric exchange energy between the acceptor’s nonbonding Natural Localized Molecular Orbital (NLMO) n and H-D bonding NLMO σ. The natural steric analysis is accessible in NBO 4.0 and later versions via the STERIC keyword.

The strength of the individual hydrogen bonds was also characterized using the QTAIM topological parameters and delocalization index. The QTAIM calculations were performed with the AIMAII [51] software.

The delocalization index (*δ*) is a descriptor capable of characterizing both closed-shell and shared-shell interactions [33]. Its value is a measure of the number of electrons delocalized between atoms. *δ*(A, B) between atoms A and B is calculated within the QTAIM theory, which defines atomic basins in a molecule. Having defined atomic basins, it is possible to calculate δ(A, B) as:

(6) $$\delta \left(A,\;B\right)=4\sum_{i,j}^{N/2}S_{\mathrm{ij}}\left(A\right)S_{\mathrm{ij}}\left(B\right),$$

where *S*_ij_(A) and *S*_ij_(B) are the overlaps between orbitals i and j in the atomic basin of A and B, respectively.

Harmonic Oscillator Model of Aromaticity (HOMA) [52] was chosen as an aromaticity descriptor. It is a geometry-based descriptor dependent on the bond lengths of the studied system in comparison to a hypothetical, fully aromatic reference system. HOMA is defined as:

(7) $$\mathrm{HOMA}=1-\frac{1}{n}\sum_{i}^{n}\alpha_{j}{\left(d_{\mathrm{opt},j}-d_{j,i}\right)}^{2},$$

where *n* is the number of bonds taken into account when carrying out the summation, j means the type of bond (e.g., CC or CN), α_j_ is an empirical normalization constant, *d*_opt,j_ is the optimal length of a given bond assumed to be realized for full aromatic systems, and *d*_j,i_ is an actual bond length in the studied system. The values of HOMA were calculated using the Multiwfn [53] program, with HOMA constants (*α*_j_ and optimal bond lengths) taken from Krygowski’s paper [54].

## 3. Results and Discussion

The discussion of the results is divided into five parts. The first four concern various aspects of the substituent effect. The last part presents the interrelationships between various characteristics describing the strength of individual hydrogen bonds. The obtained values of the substituent effect descriptors (*c*SAR, HOMA) and hydrogen bond strength parameters (*E*_HB_*, d*_HB_*, ρ*_BCP_*,* δ(H,A), ∇^2^*ρ*_BCP_, *E*_def_, *E*_SM_) for the substituted WC and HG base pairs as well as the adenine dimers are presented in Tables S1–S7 (Supplementary Materials).

### 3.1. Classical Substituent Effect-Intramolecular Interactions

**Figure 3:**
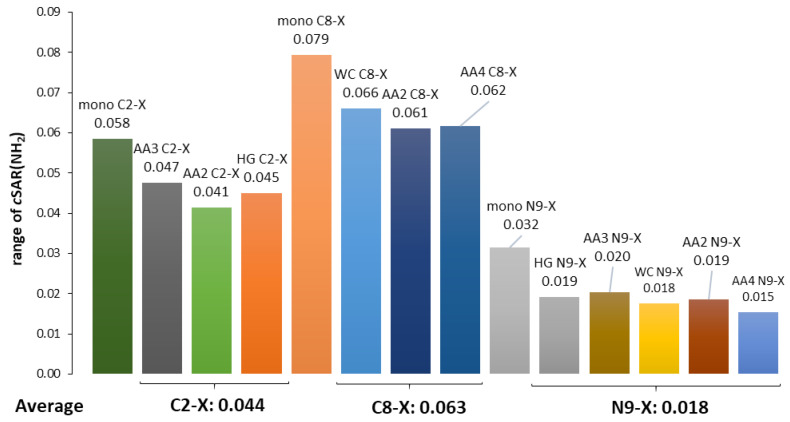
Ranges of cSAR(NH_2_) variability and their averaged values in substituted adenine monomers and dimers (WC, HG, and AA).

Adenine contains an amino group at the C6 position, which in base pairs is involved in the hydrogen bond, either through the interaction of NH**⋯**O or through N**⋯**HN, as shown in Figure 1 and Figure 2. In the studied systems, the substituent also influences the electronic properties of the amino group. The cSAR parameter shows how much these interactions affect its electronic structure. Figure 3 illustrates the ranges of the *c*SAR(NH_2_) values (their exact values are listed in Table S1 (Supplementary Materials)). In all cases, the range of the *c*SAR(NH_2_) values due to the substituent effect is always greater for the adenine monomer than its dimers, in which the -NH_2_ group is involved in H-bonding. For adenine in the AA and HG/WC pairs substituted at the C2, C8, or N9 positions, the decrease in the average value ranges compared to the monomer value is equal approximately to 76%, 80% and 56%, respectively. This shows that the H-bonding of the NH_2_ group in dimers causes its weaker propensity for the substituent effect.

The use of the classical interpretation of the substituent effect allows us to consider how the substituent can affect the properties of the reaction site; in our case, it is the adenine NH_2_ group. For this purpose, the Hammett-type linear equation is used, in which the slope (*a*, also known as the reaction constant) describes the sensitivity of the reaction site to the influence of X substituents. Thus, the electronic structure of the NH_2_ group, involved in H-bonding, is described by the dependences of *c*SAR(NH_2_) on *c*SAR(X), as presented in Table 1 and Figure S6 (Supplementary Materials). In all cases, the regressions have good or at least acceptable determination coefficients (*R*^2^ > 0.81).

Looking at the data in Table 1, some observations can be made:

1. The substituents at the C8 position in the five-membered adenine ring in the WC pair have a stronger influence on the electronic structure of NH_2_ involved in the H-bond than those attached to the C2 in the six-membered ring of the HG pair by the ratio of 0.219/0.183 = 1.20.
2. The substituents attached to N9 in both pairs, WC and HG, affect the electronic structure of NH_2_ involved in the H-bond by almost the same extent, by the ratio (WC/HG) of 1.03.
3. In all cases of adenine dimers, the effect of substitution at the C2 or C8 position is greater than that at N9 by an average ratio of 1.88.

In the first case, the number of bonds, *n*, between the substituted C atom and another one substituted by the NH_2_ group involved in H-bonding is equal to three for the WC pair, whereas for the HG pair it is equal to two. According to the documented rule, substituents affect “the reaction site” more strongly from the para position (the number of bonds between functional groups *n* = 3) than from the meta one (*n* = 2) [55,56]; our case follows this rule. For the next point (2), *n* = 3 for both cases, and hence there are almost identical substituent effects from N9 on NH_2_. Finally (3), the comparisons of the effect of substituents attached to carbon and nitrogen atoms reveal that the influence of substituent at nitrogen is significantly weaker. A possible interpretation is that the lone electron pair at the nitrogen atom can be directly involved in the interaction with the substituent (N-X), thus resulting in its weaker strength of interaction at longer distances.

### 3.2. Classical Substituent Effects-Intermolecular Interactions

The properties of the adenine amino group can also be characterized by the strength of the hydrogen bond in which it is involved. Besides this, substituents may also affect other hydrogen bonds in the system. The following descriptors were used to describe the strength of an individual hydrogen bond: its length (*d*_HB_); energy (*E*_HB_); electron density at the H-bond critical point (*ρ*_BCP_); and delocalization index, δ(H,A). Their calculated values are collected in Table S7 (Supplementary Materials).

For the WC and HG base pairs, there are three types of H-bonds (see Figure 1 and Figure S1 (Supplementary Materials)):

- (HB1) NH⋯O, in which NH of the amino group is proton-donating towards the oxygen atom of uracil;
- (HB2) N⋯HN, in which the nitrogen atom of the adenine ring is proton accepting from the NH endo group in uracil;
- (HB3) a weak CH**⋯**O, where the C-H of the adenine ring, C2-H in WC and C8-H in HG, interacts with the carbonyl group of uracil.

In the case of HB3, despite the presence of a bond critical point satisfying the Koch-Popelier [57] criteria for hydrogen bonding, an NBO analysis showed negligible interactions. For this reason, only HB1 and HB2 will be discussed.

**Figure 4:**
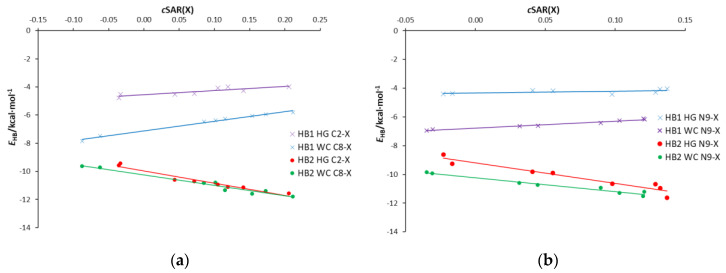
Dependence of the HB1 and HB2 energies on cSAR(X) for the WC and HG base pairs substituted at (**a**) C8 and C2 and (**b**) N9 positions of the adenine moiety.

Therefore, the following problem should be considered—how the substituents attached to C8 (WC pair), C2 (HG pair), and N9 in both pairs affect the individual H-bond stability. As above, such problems can be presented by a linear correlation of the hydrogen bond strength descriptor versus *c*SAR(X); its slope describes the sensitivity of H-bonding to the effect of the electron-accepting/donating properties of the substituent expressed by cSAR(X). Figure 4 shows the dependence of the HB1 and HB2 hydrogen bond energy on cSAR(X) for the substituted WC and HG pairs, while the slopes and determination coefficients for all the H-bond descriptors used are summarized in Table 2. A first look at its contents reveals that the slopes of the equations for the HB1 and HB2 bonds differ by a sign. Moreover, in general, regressions have at least good determination coefficients (*R*^2^ > 0.92); only in the case of the small variability of hydrogen bond descriptors are they significantly worse. In the further discussion, we consider the dependences of the HB1 and HB2 energies (*E*_HB_) on the electron-attracting/donating properties of substituents attached to C8, C2 or N9 atoms in WC, HG and AA base pairs. Alternatively, the other relationships presented in Table 2—i.e., *d*_HB_, *ρ*_BCP_, and δ(H,A) on *c*SAR(X)—lead to similar conclusions.

For HB2 in the HG and WC pairs, the slopes are −8.74 and −7.49, respectively. This indicates an increase in the hydrogen bond stability with an increase in the electron-donating power of substituents—i.e., the *c*SAR(X) values. Thus, in addition to the substituent effect on the amino group, the substituent X also affects the proton-accepting abilities of N atoms of both adenine rings. In these cases, the increase in the *c*SAR(X) values causes the proton-accepting nitrogen atoms—N1 in the WC pair and N7 in the HG pair—to increase their negative charge. This, in turn, increases the attracting forces towards the hydrogen donating NH group of uracil. A slightly higher slope for HB2 in HG than in the WC base pairs may be explained by a better delocalization way of transmission of the substituent effect. In the case of the HG base pair, the substituent is attached to C2 belonging to a fully aromatic ring, which may be represented by two equivalent canonical structures. In the case of the WC pair, only one (unexcited) canonical structure is possible, i.e., exhibits a lower aromatic character. Hence, there is a worse condition of transmission of the substituent effect.

The HB1 H-bonds represent a different situation. In both cases, an increase in the *c*SAR(X) values of substituents (i.e., their electron-donating ability) results in a decrease in the HB1 stability expressed by the slopes, which is equal to 3.01 and 6.82 for the HG and WC pairs, respectively. This difference is consistent with the observation of the substituent effect X on the properties of the NH_2_ group (Figure S6a (Supplementary Materials)), which is involved in the HB1 hydrogen bond. The electron-donating properties of the amino group decrease with the increasing electron-donating ability (more positive *c*SAR(X) value) of substituent X. Therefore, substitution at the carbon atom (C8 or C2) affects the proton-donating ability of the amino group, decreasing the *E*_HB_ for the electron-donating X groups. As mentioned above, the difference in the slopes may be explained by a well-known rule—that the substituent effect acting via three bonds (e.g., para-like interactions in benzene derivatives) is more effective than when it operates via two bonds (meta-like interactions). It is usually interpreted that the resonance effect acts more strongly through three than two bonds [55].

The stabilities of HB1 and HB2 in the WC and HG base pairs in the dependence on substitution at N9 are shown in Figure 4b. Despite a similar influence of the X substituent on the properties of the NH_2_ group (Figure S6a (Supplementary Materials)), its effect on both H-bonds is significantly different. Furthermore, trends in the effect of substituents on hydrogen bonds’ stability are similar to those observed for carbon-substituted AU pairs. The only differences are the weaker substituent effect on the HB1 bond and the stronger on the HB2 bond compared to that observed in C-X systems. For the HB1 H-bond, the obtained slopes are 4.93 and 1.34 for the WC and HG pair, respectively (6.82 and 2.71 for the C-X series). In the case of HB2, the resulting slopes are −9.66 and −14.35, respectively, while they are −7.49 and −8.74 for C-X systems, respectively. These results reveal the important role of the lone electron pair at the nitrogen (N9) atom in hydrogen bonding for AU base pairs. The substantial HB2 enhancement by the substituent effect in the HG pair, compared to the WC pair, is due to the fact that the nitrogen atom of the five-membered ring is the proton acceptor of this H-bond. This enhancement is associated with an increase in the charge of this adenine nitrogen atom interacting with the NH group of uracil. This, in turn, is due to the increasing electron-donating power of the substituent—i.e., an increase in the *c*SAR(X) values.

The results of studies on the effect of substituents attached to C8, C2 or N9 in one adenine on the stability of individual H-bonds in adenine-adenine pairs are presented in Table 3 and Figure S7 (Supplementary Materials). They reveal a similar relationship between the position of the substituent and the stability of H-bonding for adenine dimers, as observed for the WC and HG pairs. The increase in the electron-donating power of substituents causes a decrease in the HB1 stability while strengthening the second H-bond, HB2. This is documented by the positive and negative slope values (Table 3), respectively. However, for AU pairs (both WC and HG), the greater sensitivity of the substituent effect (absolute slope values, Table 2) on the H-bond stability has always been observed for HB2 compared to HB1. This does not apply to mono-substituted adenine dimers. For C8-substituted systems (AA2 and AA4), HB1 is more sensitive than HB2, while the opposite is true for C2-substituted derivatives (AA2 and AA3). This is consistent with the results shown above, where the substituents at the C8 position affect the electronic properties of the adenine amino group more than at the C2 and N9 positions (Table 1). Therefore, they cause greater changes in HB1 stability, which can be expressed by the ratio of slopes *a* in Table 3. Considering the hydrogen bonding energy, the C8/C2 ratio in AA2 pairs is 1.68, and for C8/N9 in AA4 pairs it is 1.44.

In the case of N9-substituted systems, much larger changes in HB2 strength than in HB1 were noted for the AA2 and AA3 dimers, in which the nitrogen atom of the five-membered ring (N7) is the proton acceptor of this H bond. For AA4 N9-substituted derivatives, HB1 is slightly more sensitive to the effect of substituents than HB2; the slopes are 5.128 and −4.715, respectively (Table 3). For C8 analogs, they are 7.362 and −3.998, respectively. Interestingly, these systems are characterized by the smallest effect of substituents on the electronic structure of the amino group (Table 1) and the strongest intermolecular interactions between adenine-adenine base pairs. In this case, the amino group (proton donor) and the N1 nitrogen atom, which is a proton acceptor and adjacent to the C6 carbon atom, are involved in hydrogen bonds (Figure 2c). A comparison of the structure of the N1-C6-NH_2_ part in the monomer and dimer shows a significant increase in resonance effects in this fragment. Additionally, in AA4 pairs and WC pairs, H-bonds together with six-membered pyrimidine rings form anthracene-like geometry, which results in the additional stabilization of these systems. This is confirmed by the analysis of the aromaticity of a quasi-aromatic ring consisting of H-bonds [58], which can be described using the QTAIM theory by the total electron energy density at the ring critical point (*H*_RCP_). The obtained differences between the average *H*_RCP_ values (in a.u.) in the base pairs are: ∆H(AA4 − AA2) = 0.00034, ∆H(AA4 − AA3) = 0.00061, and ∆H(WC − HG) = 0.00038; the *H*_RCP_ values for the AA4 and WC dimers are 0.00115 and 0.00135, respectively. It is therefore consistent with the fact that the quasi-aromatic hydrogen bonding systems in AA4 and WC pairs show the strongest H-bonds.

Three types of double substituted adenine-adenine pairs were considered in which the same substituent is attached to both adenines. Two of them are symmetric (AA3 C2-X, C2-X, and AA4 C8-X, C8-X) and one is asymmetric (AA2 C2-X, C8-X). The relationships between the energy of individual H-bonds and cSAR(X) are shown in Figure S8. It documents the more complex nature of mutual interactions between the substituents affecting the H-bonding in question. This is manifested by the slopes of linear equations and determination coefficients (*R*^2^). However, it is again clearly shown that stronger interactions come out from substitution at the C8 position.

### 3.3. Substituent Effect on the Transmitting Moiety

**Figure 5:**
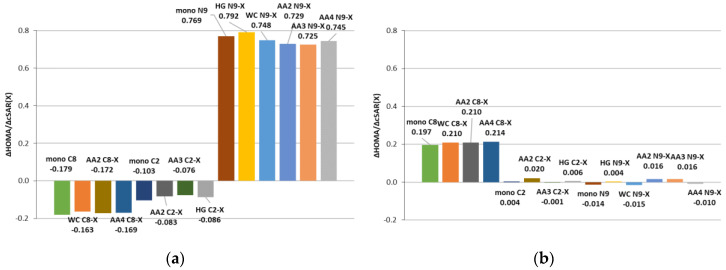
Influence of the substituents on Harmonic Oscillator Model of Aromaticity (HOMA) values for (**a**) five-membered ring and (**b**) six-membered ring in the studied base pairs, approximated by the slope ∆HOMA/∆cSAR(X) values, where ∆HOMA = HOMA(NH_2_) − HOMA(NO_2_) and ∆*c*SAR(X) = *c*SAR(NH_2_) − *c*SAR(NO_2_).

Adenine molecule consists of five-membered (AD5) and six-membered (AD6) heterocyclic rings. These rings act as transmitters of the substituent effect between X and the “reaction center”—the amino group. In the studied most stable 9H tautomer, both rings have 6π electrons, so they satisfy the Hückel’s 4n + 2 rule [59]. In the AD6 ring, each atom provides a 1π electron to the delocalized structure, while in the case of AD5, four atoms provide a 1π electron, and the N9 atom provides 2π electrons for the overall delocalization. Hence, the substituent effect on the π-electron delocalization may not be equivalent. The influence of the substituent on the electronic structure of adenine rings can be expressed using aromaticity indices. For this purpose, the HOMA index was used, which can characterize local aromaticity. The calculated HOMA index values of both adenine rings are summarized in Tables S1–S3 (Supplementary Materials), while Table S6 (Supplementary Materials) contains the HOMA values of uracil from the WC and HG pairs. The effect of substituents on HOMA for six- and five-membered adenine rings was approximated by slope values ∆HOMA/∆*c*SAR(X), where ∆HOMA = HOMA(NH_2_) − HOMA(NO_2_), and ∆*c*SAR(X) = *c*SAR(NH_2_) − *c*SAR(NO_2_). The obtained results for the studied base pairs and monomers are shown in Figure 5.

The differences in the HOMA values between the substituted and unsubstituted adenine molecules (Tables S8 and S9 (Supplementary Materials)) show that the substitution at C8 affects the electronic structure of the AD6 ring more than the C2 and N9 substitution. Besides, for the C8 substitution, as the electron-donating effect of the substituent described by *c*SAR(X) increases, the HOMA value of the AD6 ring also increases. Moreover, the intermolecular interactions in pairs slightly enhance the effect of substitution from the C8 position on the aromaticity of the six-membered ring compared to the monomer. In the case of C2 and N9 substitution, HOMA does not change monotonically with *c*SAR(X).

The aromaticity of the AD5 ring of adenine has the highest sensitivity to N9 substitution, and the HOMA values increase with the electron-donating ability of the substituent. This can be explained by an interaction between the substituent at the N9 position and the lone electron pair of the N9 atom, which is important for the delocalization in the AD5 ring. An electron-accepting group, such as NO_2_, may withdraw electrons from the N9 lone electron pair and disturb electron delocalization within the AD5 ring. The substitution at positions C8 and C2 causes smaller changes in the AD5 HOMA value, although in these cases the effect is opposite—the HOMA value decreases with the increasing electron-donating power of the substituent, i.e., *c*SAR(X) value.

Furthermore, in WC and HG pairs, the substituents in adenine also affect the aromaticity of uracil (Table S6 (Supplementary Materials)). Again, the strongest effect is observed from the position C8 than from N9, and the smallest from the position C2. Therefore, it can be concluded that long-distance substituent effects are documented.

### 3.4. Reverse Substituent Effect

**Figure 6:**
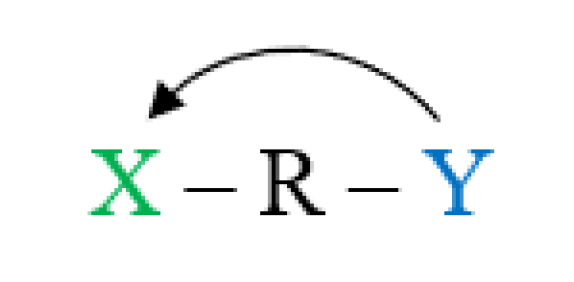
Reverse substituent effect in the X-R-Y system.

The reverse substituent effect quantitatively describes how substituted moiety affects the electronic properties of the substituent X in a system such as R-X or, more generally, X-R-Y. Schematically, it may be presented as in Figure 6.

It should be emphasized that the reverse substituent effect has been known from the beginning, hence there are different substituent constants σ for the para and meta positions [34], similarly to substituent constants σ^+^ and σ^−^ for electron-accepting and -donating reaction sites, respectively [60]. The use of *c*SAR(X) allows a quantitative description of the electron-donating or accepting properties of the substituent in any system. This gives additional insight into understanding the substituent effect dependent on various substituted moieties.

**Figure 7:**
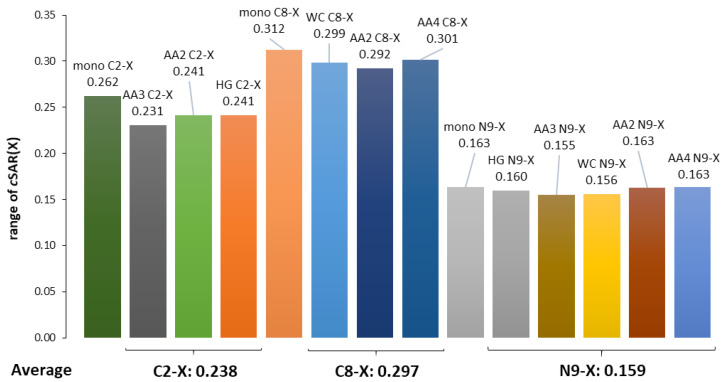
Ranges of *c*SAR(X) variability and their averaged values for substituents attached to C2, C8, and N9 in adenine monomer and its pairs (WC, HG, and adenine-adenine).

In our case, we consider how the amino group, variously involved in H-bonding, affects the properties and electronic structure of the substituents expressed by *c*SAR(X) values. Figure 7 presents the ranges of *c*SAR(X) values in the dependence on the position and type of moieties to which they are attached. All the data for dimers are compared with the values for adenine monomers.

A comparison of the ranges of *c*SAR(X) values in the adenine monomer and its pairs (WC, HG, and AA) leads to the conclusion that, in the adenine monomer, these ranges are greater than in its pairs for substitution at carbon atoms and almost comparable for substitution at nitrogen. In the case of substitution at C2, the ranges are averaged to 0.238, a value that is significantly lower than for the series substituted at C8 (mean range 0.297), with the ratio of C8/C2 = 1.25. The explanation of this is the same as formerly: the number of bonds between C8, C2 and C6 (with NH_2_ group attached) is 3 and 2, respectively. In the case of substitution at the nitrogen atom, the former conclusion that the lone electron pair causes a weaker substituent effect due to its possible direct interaction with the substituent can be repeated.

The ranges of *c*SAR(X) change for the examined substituents attached to the C8, C2 and N9 positions, and more generally the ranges for those attached to the carbon and nitrogen atom in adenine for the studied systems are shown in Figure S9 (Supplementary Materials). Changes in these ranges for a given substituent depend on the substitution position (C2, C8, and N9). In the extreme case (N9-Cl), the variation range is 0.042/0.160—i.e., 26% and slightly less for carbon substitution (NO, 0.066/0.272—i.e., 24%).

### 3.5. Interrelations between Hydrogen Bond Parameters

As shown above, substituents can significantly change the strength of intermolecular interactions. The presence of a substituent in the adenine molecule affects hydrogen bonds in base pairs, either strengthening or weakening them. The strength of individual H-bonds has been characterized by several descriptors, such as the energy of the H-bond (calculated according to the NBO approach), *E*_HB_; its length, *d*_HB_; and the QTAIM parameters—i.e., the electron density at the bond critical point, *ρ*_BCP_; its Laplacian, ∇^2^*ρ*_BCP_; and the delocalization index between hydrogen and H-bond acceptor atom A, δ(H,A). Thus, the results for the N⋯H and O⋯H hydrogen bonds in the studied dimers can be used to check the interrelationships of these descriptors of intermolecular interactions in the obtained range of their changes. The values of all applied characteristics are collected in Table S7 (Supplementary Materials).

In general, hydrogen bonds can be classified as weak, moderate, and strong. Jeffrey [61] distinguishes weak H-bonds as those for which the range of absolute energy value is 1–4 kcal/mol; for moderate H-bonds, these energies are 4–15 kcal/mol, and they are 15–40 kcal/mol for strong hydrogen bonds. However, it should be emphasized that there are no “sharp” borders between these hydrogen bonds [62]. The QTAIM theory is also a source of energetic parameters. Rozas, Alkorta, and Elguero [63] suggested that the Laplacian as well as the total electron energy density at hydrogen bond BCP, *H*_BCP_, should both be used as criteria to characterize hydrogen bonding. They proposed for weak H-bonds that both ∇^2^*ρ*_BCP_ and *H*_BCP_ > 0; for medium H-bonds, they are ∇^2^*ρ*_BCP_ > 0 and *H*_BCP_ < 0, while for strong ones, both ∇^2^*ρ*_BCP_ and *H*_BCP_ < 0. The topological QTAIM parameters also provide information on the nature of the interaction [64,65]. It has been shown that H-bonds shorter than 1.2 Å exhibit a covalent nature, bond lengths in the range 1.2–1.8 Å are associated with the partially covalent character, and H-bonds longer than 1.8 Å are noncovalent; this is also referred to as shared-shell, intermediate closed-shell, and closed-shell, respectively.

**Figure 8:**
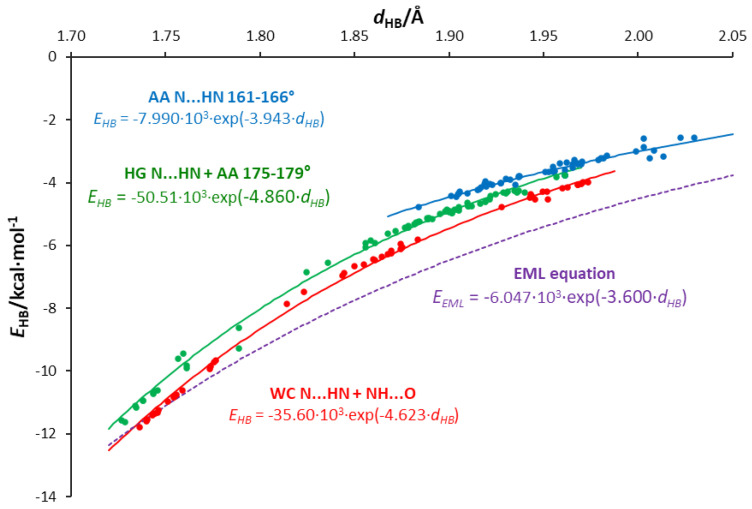
Dependences of the H-bond energies, *E*_HB_, on their lengths, *d*_HB_, in the studied adenine-uracil and adenine-adenine base pairs.

All the calculated energies and their corresponding H-bond lengths in the AU and AA pairs are shown in Figure S10 (Supplementary Materials). The linear equation of ln(|*E*_HB_|) against the H-bond lengths (Figure S11 (Supplementary Materials)) indicates the exponential nature of the relationship shown in Figure S10 (Supplementary Materials). However, a deeper look at both figures indicates that three groups of H-bonds should be distinguished from all points, as shown in Figure 8. The first group (in red) contains O⋯H and HB2 (N**⋯**H) interactions in the WC pairs, the second (in green) contains HB2 in HG pairs and almost linear H-bonds (angle > 175°) in adenine dimers, while the third group (in blue) contains bonds with an H**⋯**N-H angle between 161° and 166°; this division is justified by the linear relations shown in Figure S12 (Supplementary Materials). For the same H-bond length, the strength of the interaction decreases as the group number increases. Interestingly, in the case of the strongest interactions, both the nitrogen and oxygen atoms act as acceptors of H-bond protons. Thus, the same relationship describes two types of hydrogen bonds. For comparison, a curve representing the EML equation [24] is added in Figure 8. This equation is based on the exponential fit for 83 experimentally observed in O⋯H hydrogen bonds.

Hydrogen bonds in AU base pairs are much stronger than in AA dimers (Table S7 (Supplementary Materials)). In the case of AU systems, N⋯H interactions (HB2) are stronger than O⋯H (HB1) H-bonds. For the same H-bond length, HB2 is slightly stronger in WC than the HG series. The HB1 bonds in WC pairs are much shorter than in HG dimers, and therefore significantly stronger. For AA dimers, the strength of interactions depends on the N⋯H-N angle, and, as expected, the linear hydrogen bonds are stronger (Figure 8). Additionally, the obtained values of ∇^2^*ρ*_BCP_ and *H*_BCP_ for HB2 H-bonds (N⋯H) in the AU base pairs and the strongest AA dimers’ H-bonds—AA4 C8-X and AA4 N9-X, X = NO, NO_2_ (∇^2^*ρ*_BCP_ > 0 and *H*_BCP_ < 0, blue points in Figure S13 (Supplementary Materials))—reveal the partially covalent nature of these interactions. H-bonds in AA4 dimers are longer than 1.8 Å (in the range 1.82–1.88 Å). For the other hydrogen bonds, both QTAIM descriptors are positive, indicating the closed-shell nature of the interactions.

**Figure 9:**
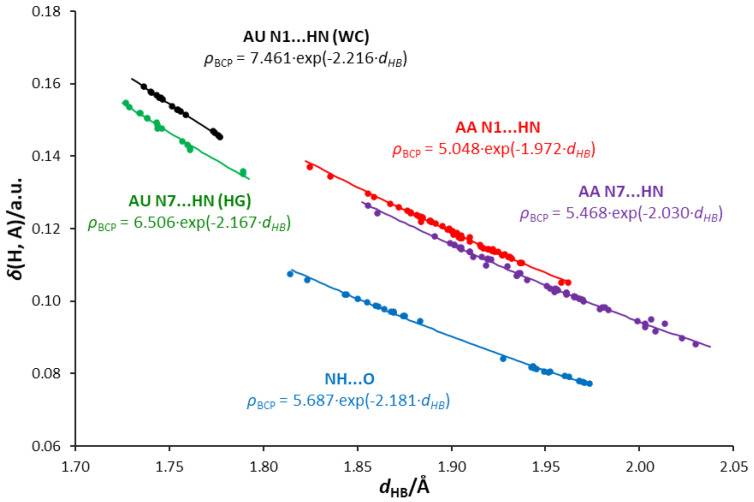
Dependences of the delocalization index, δ(H,A), on the H-bond lengths, *d*_HB_, in the studied adenine-uracil and adenine-adenine base pairs.

The relationships between the electron density at the H-bond critical point, *ρ*_BCP_, or delocalization index, δ(H,A), and the H-bond length are shown in Figure S14 (Supplementary Materials) and Figure 9, respectively. The exponential nature of these relationships is confirmed by the linear equations ln(*ρ*_BCP_) and ln(δ(H,A)) in relation to the H-bond length, shown in Figures S15 and S16 (Supplementary Materials). The use of these electronic hydrogen bond descriptors allows us to distinguish three types of interaction:

- NH⋯O, where the oxygen atom of the uracil molecule is the H-bond acceptor and adenine amino group is the H-bond donor.
- N1⋯HN, where the N1 acceptor atom is a part of a six-membered adenine ring.
- N7⋯HN, where the N7 acceptor atom is a part of a five-membered adenine ring.

**Figure 10:**
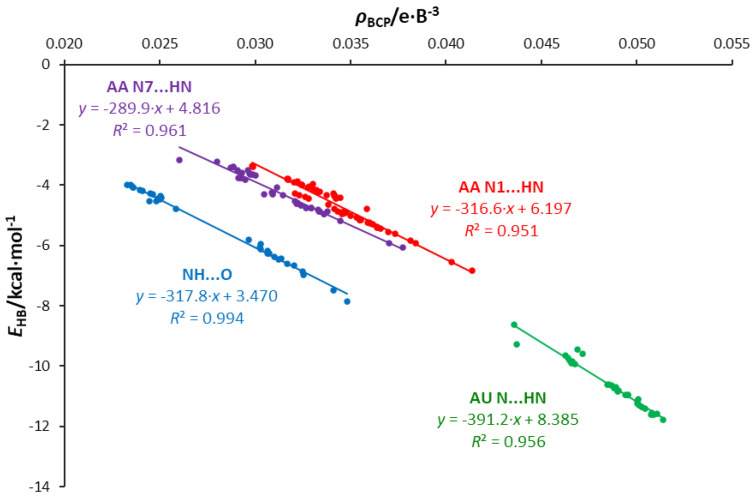
Adenine-adenine dimers. Energy of individual H-bonds as a function of the electron density at the H-bond critical point *ρ*_BCP._

For these interaction groups, the dependence of the H-bond energy on the electron density at its BCP is shown in Figure 10. In the case of N1⋯HN, energies corresponding to a given *ρ*_BCP_ value are higher than for N7⋯HN; the same applies to the electron density at BCP for a given hydrogen bond length (Figure S14 (Supplementary Materials)).

Figure 9 shows an interesting relationship between the delocalization index δ(H,A) and the H-bond length *d*_HB_. The values of δ(H,A) for a given *d*_HB_ are higher for the N1⋯HN-type bonds than for N7⋯HN in the case of both AA and AU pairs. This shows that N1⋯HN bonds have a higher covalent character than N7⋯HN bonds, which is consistent with the values of ∇^2^*ρ*_BCP_ and *H*_BCP_ descriptors discussed earlier and presented in Figure S13 (Supplementary Materials). Even lower values of *δ*(H,A) are seen for the NH⋯O-type AU HB1 bonds (marked in blue). This may be interpreted by a stronger attraction of electrons by more electron-attracting oxygen than nitrogen atoms in these interactions, and, therefore, the less covalent character of the NH⋯O hydrogen bond compared to N⋯HN. Thus, the relationships for N⋯H bonds depend on both the type of the acceptor atom (N1 or N7) and the nature of the interactions. This also confirms the interrelationship between the delocalization index and the electron density at BCP of the H-bond (Figure S17 (Supplementary Materials)).

## 4. Conclusions

This theoretical study provides insight into the structural consequences of the substituent effect in biologically important adenine dimers. Four specific aspects were considered: classical SE on intra- and intermolecular interactions, effects on the transmitting moiety, and reverse SE. Additionally, the interrelations between hydrogen bond parameters are presented, revealing more information about the nature of H-bonding in adenine base pairs and other heterocyclic systems.

The classical SE on intramolecular interactions, described by the *c*SAR index, shows that the substituent has a diversified effect on the amino group, and this effect depends on its position. The transmission of the SE to the amino group from the C8 position is more effective than from the C2 or N9 positions. These differences can be explained by an analogy with benzene substituted in the para and meta positions. The C8 position in the adenine molecule is *n* = 3 bonds away (para-like effect), while C2 is *n* = 2 bonds away from the amino group (meta-like effect). N9 substitution has the weakest effect on the amino group due to an interaction of the substituent and N9 atom lone electron pair. These effects also explain the classical SE on intermolecular interactions—H-bonds. They are presented as relations between the cSAR(X) and H-bond energy, length, and H-bond critical point parameters. The H-bond formed by the substituted adenine acceptor atom shows more sensitivity to SE transmitted from N9, which is caused either by the close distance between the N9 and N7 atoms or by the effect on the lone electron pair of the N9 atom.

Changes in the HOMA index for adenine rings show that a substituent, depending on its position, may have either a strong or almost negligible effect on the aromaticity. The five-membered ring aromaticity is highly influenced by N9 because of an interaction between the substituent and the N9 lone electron pair, which greatly contributes to the ring’s delocalized structure. The changes in the six-membered ring aromaticity caused by the substituent at the C8 position of the five-membered ring reveal an interesting long-distance SE.

The reverse SE is an effect of the amino group involved in varying intermolecular interactions on the attached substituent. The results presented as changes in the cSAR(X) values showed that the reverse SE is weaker when the amino group forms an H-bond (in base pairs) than in monomers. Moreover, its strength is consistent with para and meta-like effects in classical SE. Furthermore, the changes in the cSAR ranges for a given substituent may reach ca. 25% of the average variation in the *c*SAR(X) range for a particular substitution position (C-X or N-X).

A deeper analysis of the hydrogen bonds and interrelations between their parameters leads to the following observations:

- Hydrogen bond energies obtained by the NBO approach are smaller for a given length than those predicted by the EML equation for systems with an O⋯H single hydrogen bond. An exponential relation between the energy and H-bond length is proposed.
- The values of energy are dependent on the H-bond angle forced by the base pair geometry. The most favorable geometry is an anthracene-like system with two six-membered rings connected by two H-bonds. Base pairs of this geometry show the strongest H-bonding.
- Some of the H-bonds exhibit a partially covalent character, confirmed by positive values of the Laplacian and negative values of the total energy density at the bond critical point.
- Relations between the QTAIM parameters differentiate the type of acceptor atom—i.e., adenine N1, N7 atoms, and the uracil O carbonyl atom. The N1 acceptor atom of the six-membered ring forms H-bonds of higher covalent character than the N7 five-membered ring. Bonds formed by the carbonyl oxygen of uracil are less covalent in character, possibly due to the higher electronegativity and attraction of electrons.

## Figures and Tables

**Table 1:** Slopes of the obtained linear dependences *c*SAR(NH_2_) vs. *c*SAR(X) and determination coefficients (*R*^2^) for the substituted WC and HG base pairs, adenine dimers, and substituted monomers (marked in **bold**, data taken from Ref. [19]) (A-C2-X, A-C8-X, A-N9-X). For asymmetrically substituted AA2 C2-X, C8-X dimer, the cSAR values of underlined monomers are taken.

	*a*	*R* ^2^
**A-C2-X**	−0.222	0.913
**HG C2-X**	−0.183	0.882
**AA2 C2-X**	−0.163	0.838
**AA3 C2-X**	−0.184	0.824
**A-C8-X**	−0.297	0.989
**WC C8-X**	−0.219	0.991
**AA2 C8-X**	−0.209	0.983
**AA4 C8-X**	−0.167	0.875
**A-N9-X**	−0.166	0.902
**HG N9-X**	−0.084	0.817
**WC N9-X**	−0.087	0.835
**AA2 N9-X**	−0.100	0.883
**AA3 N9-X**	−0.110	0.808
**AA4 N9-X**	−0.079	0.813
**AA2 C2-X, C8-X**	−0.176	0.890
**AA2 C2-X, C8-X**	−0.201	0.947
**AA3 C2-X, C2-X**	−0.182	0.818
**AA4 C8-X, C8-X**	−0.236	0.994

**Table 2:** Slopes of the obtained linear relations between the H-bond descriptors (y) and *c*SAR(X), and the determination coefficients (*R*^2^) for the substituted WC and HG base pairs.

y = *a*∙*c*SAR(X) + *b*		y = *E*_HB_		y = *d*_HB_		y = *ρ*_BCP_		y *= δ*(H,A)	
Base Pair	HB	*a*	*R* ^2^	*a*	*R* ^2^	*a*	*R* ^2^	*a*	*R* ^2^
**HG C2-X**	1	3.013	0.738	0.137	0.521	−0.00757	0.526	−0.0215	0.566
	2	−8.741	0.951	−0.134	0.982	0.0172	0.984	0.0476	0.986
**WC C8-X**	1	6.818	0.986	0.233	0.985	−0.0175	0.980	−0.0446	0.981
	2	−7.487	0.966	−0.143	0.968	0.0179	0.967	0.0485	0.968
**HG N9-X**	1	1.344	0.314	0.100	0.329	−0.00620	0.400	−0.0179	0.455
	2	−14.35	0.921	−0.341	0.964	0.0402	0.971	0.104	0.980
**WC N9-X**	1	4.927	0.971	0.182	0.929	−0.0133	0.932	−0.0343	0.934
	2	−9.655	0.967	−0.194	0.972	0.0240	0.973	0.0645	0.970

**Table 3:** Slopes of the obtained linear relations between the H-bond descriptors (y) and *c*SAR(X), and the determination coefficients (*R*^2^) for the substituted adenine dimers.

y = *a*∙*c*SAR(X) + *b*		y = *E*_HB_		y = *d*_HB_		y = *ρ*_BCP_		y = δ(H, A)	
Base Pair	HB	*a*	*R^2^*	*a*	*R^2^*	*a*	*R^2^*	*a*	*R^2^*
**AA2 C2-X**	1	3.589	0.811	0.200	0.825	−0.0156	0.825	−0.0426	0.835
	2	−3.794	0.987	−0.193	0.993	0.0152	0.994	0.0439	0.994
**AA2 C8-X**	1	6.036	0.987	0.260	0.992	−0.0218	0.985	−0.0571	0.986
	2	−3.036	0.963	−0.161	0.958	0.0126	0.965	0.0369	0.964
**AA3 C2-X**	1	2.172	0.433	0.137	0.223	−0.0096	0.262	−0.0261	0.291
	2	−4.843	0.762	−0.276	0.579	0.0184	0.610	0.0547	0.672
**AA4 C8-X**	1	7.362	0.990	0.284	0.993	−0.0252	0.989	−0.0426	0.835
	2	−3.998	0.976	−0.189	0.976	0.0155	0.974	0.0439	0.994
**AA2 N9-X**	1	1.925	0.472	0.122	0.687	−0.0096	0.712	−0.0267	0.718
	2	−6.930	0.978	−0.370	0.986	0.0276	0.975	0.0834	0.968
**AA3 N9-X**	1	1.187	0.187	0.097	0.501	−0.0068	0.575	−0.0187	0.491
	2	−5.778	0.792	−0.413	0.943	0.0261	0.931	0.0831	0.908
**AA4 N9-X**	1	5.128	0.963	0.220	0.960	−0.0190	0.964	−0.0495	0.961
	2	−4.715	0.949	−0.225	0.967	0.0185	0.967	0.0529	0.968

## Supplementary Materials

The following are available online at https://www.mdpi.com/1420-3049/25/16/3688/s1: Figure S1. Studied substituted adenine-uracil base pairs; Figure S2. Studied substituted adenine-adenine AA2 base pairs. Figure S3. Studied substituted adenine-adenine AA3 base pairs; Figure S4. Studied substituted adenine-adenine AA4 base pairs; Figure S5. Linear regressions between cSAR(X) values calculated by Hirshfeld (Hir) method and data from the VDD and NBO approaches for derivatives of the WC base pair substituted in positions C8-X and N9-X by X = NO, NO_2_, Cl, F, H, Me, OH, NH_2_; Figure S6. Relationships between cSAR(NH_2_) and cSAR(X) in substituted adenine-uracil and adenine-adenine systems; Figure S7. Dependence of HB1 and HB2 energies on cSAR(X) for adenine dimers substituted in C8, C2 and N9 positions of one adenine moiety; Figure S8. Energy of individual H-bonds as a function of cSAR(X) for adenine-adenine pairs with the same substituents attached to both adenines; Figure S9. Ranges of cSAR(X) changes for the examined X substituent attached to C2, C8, and the nitrogen (N9) and carbon atoms in the adenine monomer and its pairs (WC, HG, and adenine-adenine); Figure S10. Dependence of H-bond energies on their lengths for all hydrogen bonds in studied adenine-uracil and adenine-adenine base pairs; Figure S11. Relationship between ln(*E*_HB_) and H-bond lengths for all hydrogen bonds in studied adenine-uracil and adenine-adenine base pairs; Figure S12. Relationships between ln(|*E*_HB_|) and hydrogen bond lengths for the groups shown in Figure 8; Figure S13. Dependence of Laplacian electron density on the length of the H-bond for all hydrogen bonds in studied adenine-uracil and adenine-adenine base pairs; Figure S14. Dependences of electron density at the H-bond critical points on their lengths in the studied adenine-uracil and adenine-adenine base pairs; Figure S15. Relationships between ln(ρBCP) and H-bond lengths for all hydrogen bonds in studied adenine-uracil and adenine-adenine base pairs; Figure S16. Relationships between ln(δ(H,A)) and H-bond lengths, for all hydrogen bonds in studied adenine-uracil and adenine-adenine base pairs; Figure S17. Delocalization index δ(H,A) between H and A atoms, where A is the acceptor of H-bond as a function of electron density at H-bond critical point for AU and AA dimers; Table S1. Values of cSAR(X), cSAR(NH_2_) and HOMA for substituted adenine-uracil Hoogsteen (HG) and Watson-Crick (WC) pairs. HOMA values of five-membered adenine ring (HOMA AD5) and six-membered adenine ring (HOMA AD6); Table S2. Values of cSAR(X), cSAR(NH2) and HOMA for substituted adenine-adenine AA2 and AA3 pairs. HOMA values of five-membered adenine ring (HOMA AD5) and six-membered adenine ring (HOMA AD6); Table S3. Values of cSAR(X), cSAR(NH_2_) and HOMA for substituted adenine-adenine AA4 pairs. HOMA values of five-membered adenine ring (HOMA AD5) and six-membered adenine ring (HOMA AD6); Table S4. Values of cSAR(X) and cSAR(NH_2_). Symmetrically substituted adenine-adenine AA3 and AA4 pairs; Table S5. Values of cSAR(X) and cSAR(NH_2_). Asymmetrically substituted adenine-adenine AA2 pairs; Table S6. HOMA values of uracil ring in adenine-uracil Hoogsteen (HG) and Watson-Crick (WC) pairs with substituents at C2, C8, N9 position of adenine moiety; Table S7. Calculated hydrogen bond parameters of all studied base pairs; Table S8. Changes in aromaticity expressed by the HOMA index for (a) AD6 and (b) AD5 rings due to substitution in WC and HG pairs; Table S9. Changes in aromaticity expressed by the HOMA index for AD6 and AD5 rings due to substitution in AA dimers.
