# Supplementary material for: Mutual Relations between Substituent Effect, Hydrogen Bonding, and Aromaticity in Adenine-Uracil and Adenine-Adenine Base Pairs
Source: Molecules. 2020 Aug 13;25(16):3688. doi: 10.3390/molecules25163688 (PMC7464026; doi:10.3390/molecules25163688)
Supplement: Supplementary file 1 [file molecules-25-03688-s001.pdf]

Mutual relations between substituent effect, hydrogen bonding and aromaticity in adenine-uracil and adenine-adenine base pairs

Supplementary Materials

Paweł A. Wieczorkiewicz [1], Halina Szatylowicz [1,*] and Tadeusz M. Krygowski [2]

[1] Faculty of Chemistry, Warsaw University of Technology, Noakowskiego 3, 00-664 Warsaw, Poland
[2] Faculty of Chemistry, University of Warsaw, Pasteura 1, 02-093 Warsaw, Poland
[*] Correspondence: halina@ch.pw.edu.pl

Contents

Figure S1. Studied substituted adenine-uracil base pairs 2

Figure S2. Studied substituted adenine-adenine AA2 base pairs 2

Figure S3. Studied substituted adenine-adenine AA3 base pairs 3

Figure S4. Studied substituted adenine-adenine AA4 base pairs 3

Figure S5. Linear regressions between cSAR(X) values calculated by Hirshfeld (Hir) method and data from the VDD and NBO approaches for derivatives of the WC base pair substituted in positions C8-X and N9-X by X = NO, NO_2, Cl, F, H, Me, OH, NH_2. 4

Figure S6. Relationships between *c*SAR(NH_2) and *c*SAR(X) in substituted adenine-uracil and adenine-adenine systems. ... 5

Figure S7. Dependence of HB1 and HB2 energies on *c*SAR(X) for adenine dimers substituted in C8, C2 and N9 positions of one adenine moiety. 6

Figure S8. Energy of individual H-bonds as a function of *c*SAR(X) for adenine-adenine pairs with the same substituents attached to both adenines. 7

Figure S9. Ranges of cSAR(X) changes for the examined X substituent attached to C2, C8, and the nitrogen (N9) and carbon atoms in the adenine monomer and its pairs (WC, HG, and adenine-adenine). 7

Figure S10. Dependence of H-bond energies on their lengths for all hydrogen bonds in studied adenine-uracil and adenine-adenine base pairs. 8

Figure S11. Relationship between ln(E_{HB}) and H-bond lengths for all hydrogen bonds in studied adenine-uracil and adenine-adenine base pairs. 8

Figure S12. Relationships between ln(|E_{HB}|) and hydrogen bond lengths for the groups shown in Figure 8 9

Figure S13. Dependence of Laplacian electron density on the length of the H-bond for all hydrogen bonds in studied adenine-uracil and adenine-adenine base pairs. 9

Figure S14. Dependences of electron density at the H-bond critical points on their lengths in the studied adenine-uracil and adenine-adenine base pairs. 10

Figure S15. Relationships between ln(ρ_{BCP}) and H-bond lengths for all hydrogen bonds in studied adenine-uracil and adenine-adenine base pairs. 10

Figure S16. Relationships between ln(δ(H,A)) and H-bond lengths, for all hydrogen bonds in studied adenine-uracil and adenine-adenine base pairs. 11

Figure S17. Delocalization index δ(H,A) between H and A atoms, where A is the acceptor of H-bond as a function of electron density at H-bond critical point for AU and AA dimers. 11

Table S1. Values of cSAR(X), cSAR(NH_2) and HOMA for substituted adenine-uracil Hoogsteen (HG) and Watson-Crick (WC) pairs. HOMA values of five-membered adenine ring (HOMA AD5) and six-membered adenine ring (HOMA AD6). 12

Table S2. Values of cSAR(X), cSAR(NH_2) and HOMA for substituted adenine-adenine AA2 and AA3 pairs. HOMA values of five-membered adenine ring (HOMA AD5) and six-membered adenine ring (HOMA AD6). 13

Table S3. Values of cSAR(X), cSAR(NH_2) and HOMA for Substituted adenine-adenine AA4 pairs. HOMA values of five-membered adenine ring (HOMA AD5) and six-membered adenine ring (HOMA AD6). 14

Table S4. Values of cSAR(X) and cSAR(NH_2). Symmetrically substituted adenine-adenine AA3 and AA4 pairs. 14

Table S5. Values of cSAR(X) and cSAR(NH_2). Asymmetrically substituted adenine-adenine AA2 pairs. 15

Table S6. HOMA values of uracil ring in adenine-uracil Hoogsteen (HG) and Watson-Crick (WC) pairs with substituents at C2, C8, N9 position of adenine moiety. 15

Table S7. Calculated hydrogen bond parameters of all studied base pairs. 16

Table S8. Changes in aromaticity expressed by the HOMA index for (a) AD6 and (b) AD5 rings due to substitution in WC and HG pairs. 24

Table S9. Changes in aromaticity expressed by the HOMA index for (a) AD6 and (b) AD5 rings due to substitution in AA dimers. 25

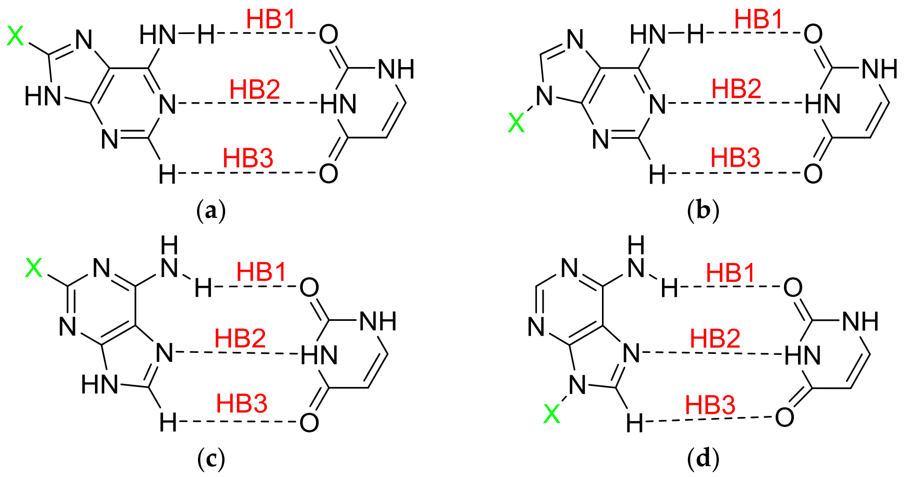

Figure S1. Studied substituted adenine-uracil base pairs: (**a**) WC C8-X, (**b**) WC N9-X, (**c**) HG C2-X, (**d**) HG N9-X.

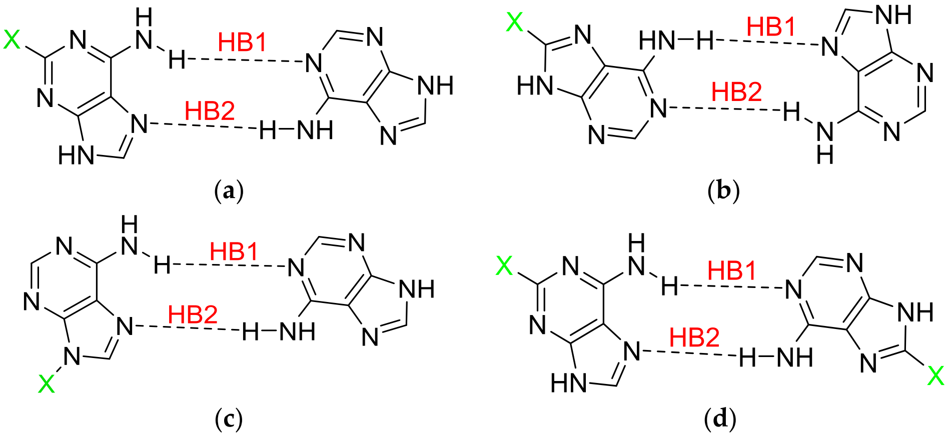

Figure S2. Studied substituted adenine-adenine AA2 base pairs: (**a**) AA2 C2-X, (**b**) AA2 C8-X, (**c**) AA2 N9-X, (**d**) double substituted AA2 C2-X, C8-X.

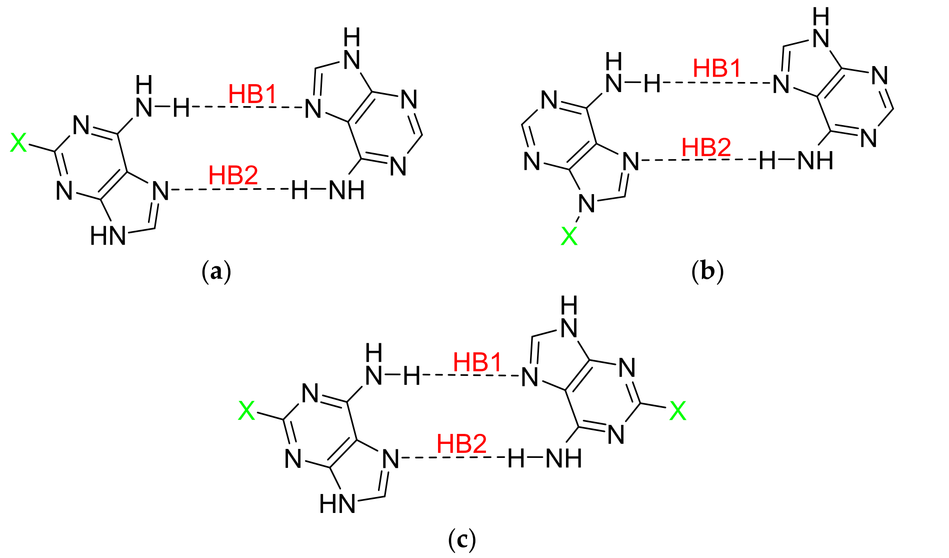

Figure S3. Studied substituted adenine-adenine AA3 base pairs: (**a**) AA3 C2-X, (**b**) AA3 N9-X, (**c**) double substituted AA3 C2-X, C2-X.

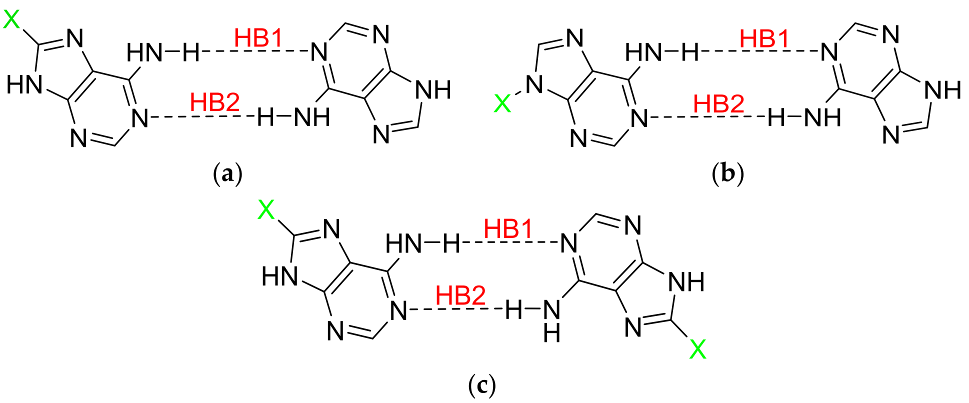

Figure S4. Studied substituted adenine-adenine AA4 base pairs: (a) AA4 C8-X, (b) AA4 N9-X, (c) double substituted AA4 C8-X, C8-X.

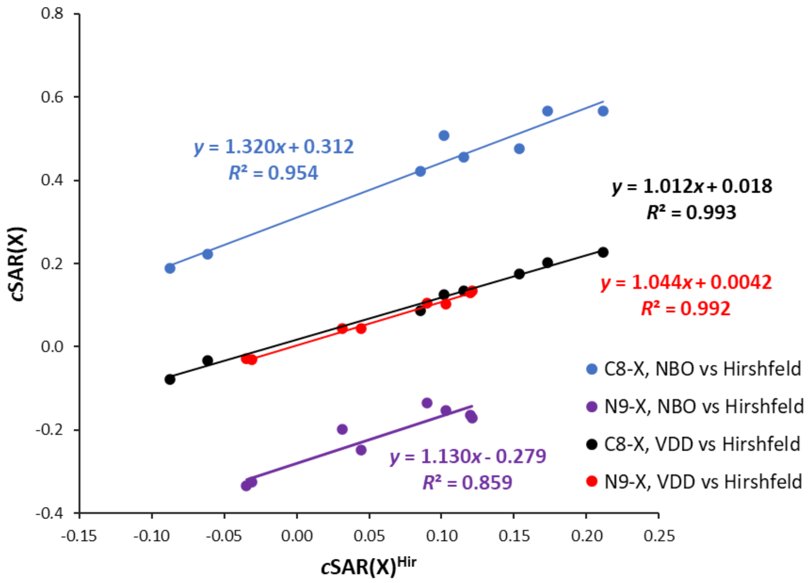

Figure S5. Linear regressions between cSAR(X) values calculated by Hirshfeld (Hir) method and data from the VDD and NBO approaches for derivatives of the WC base pair substituted in positions C8-X and N9-X by X = NO, NO_2, Cl, F, H, Me, OH, NH_2.

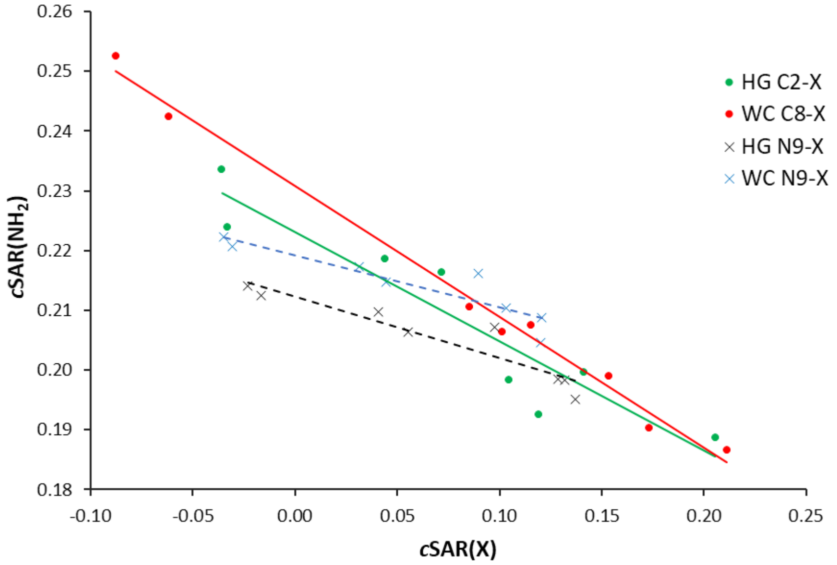

(**a**)

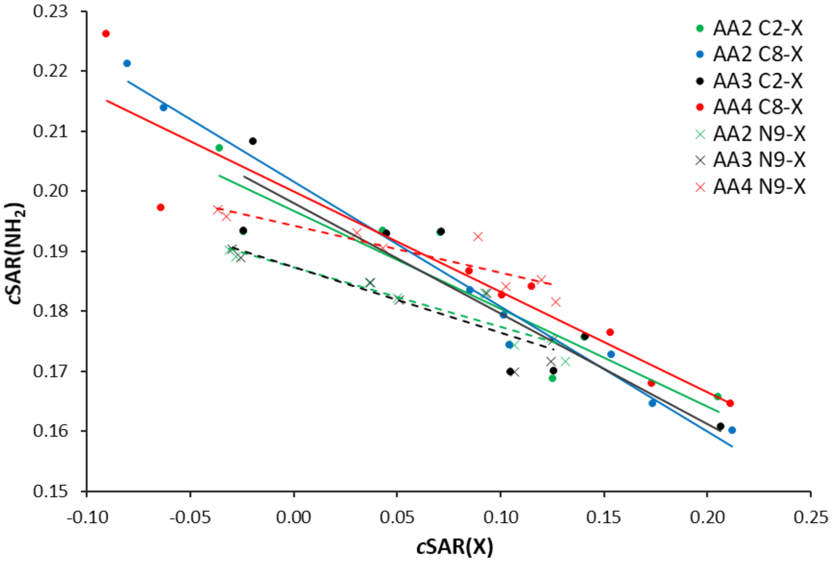

(**b**)

Figure S6. Relationships between *c*SAR(NH_2) and *c*SAR(X) in substituted adenine-uracil (**a**) and adenine-adenine (**b**) systems.

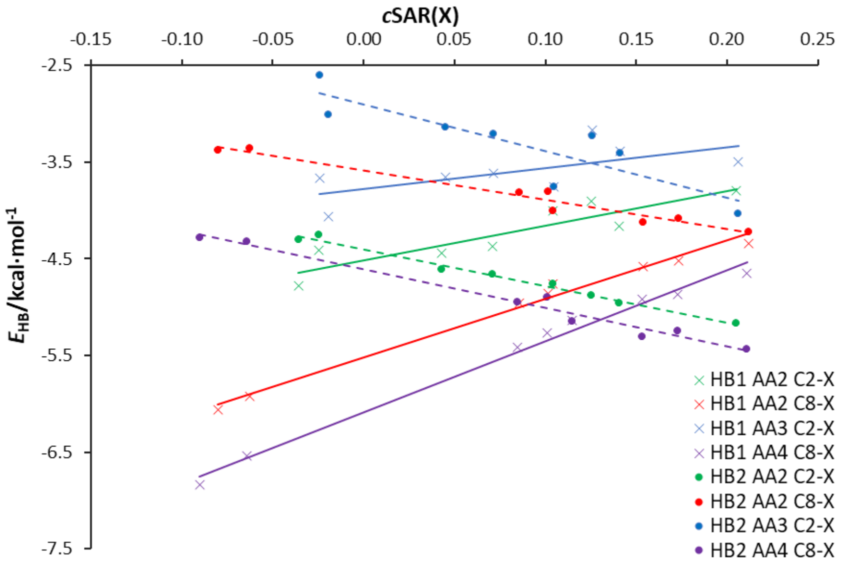

(**a**)

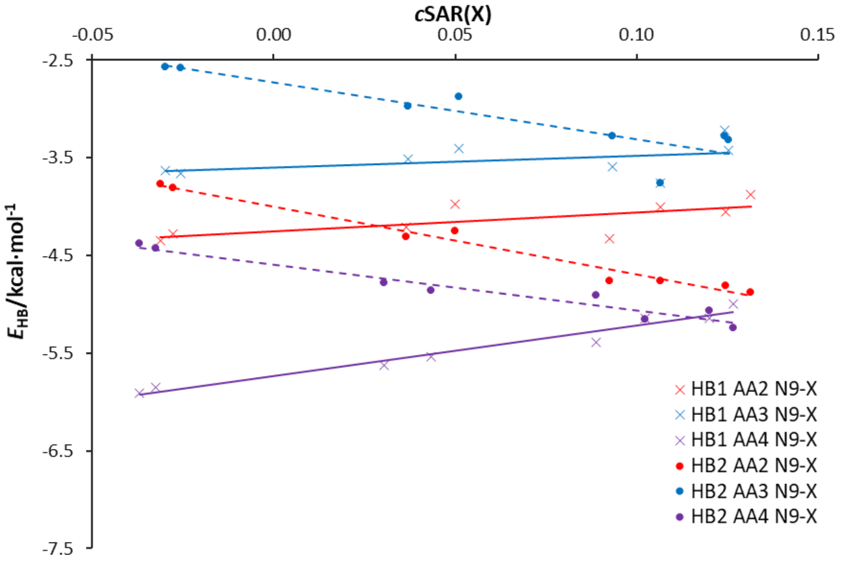

(**b**)

Figure S7. Dependence of HB1 and HB2 energies on *c*SAR(X) for adenine dimers substituted in (a) C8 and C2 and (b) N9 positions of one adenine moiety. Parameters of linear regression equations are presented in Table 3.

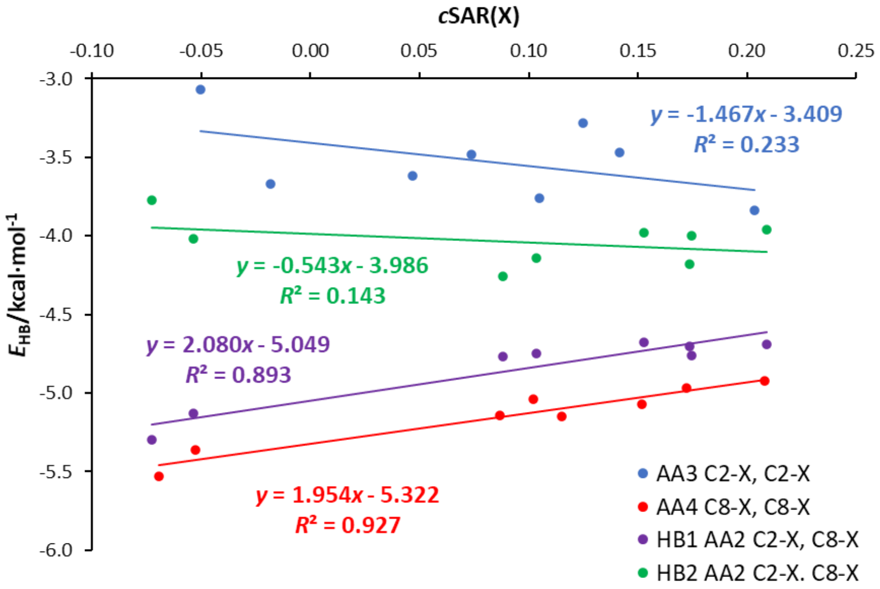

Figure S8. Energy of individual H-bonds as a function of *c*SAR(X) for adenine-adenine pairs with the same substituents attached to both adenines.

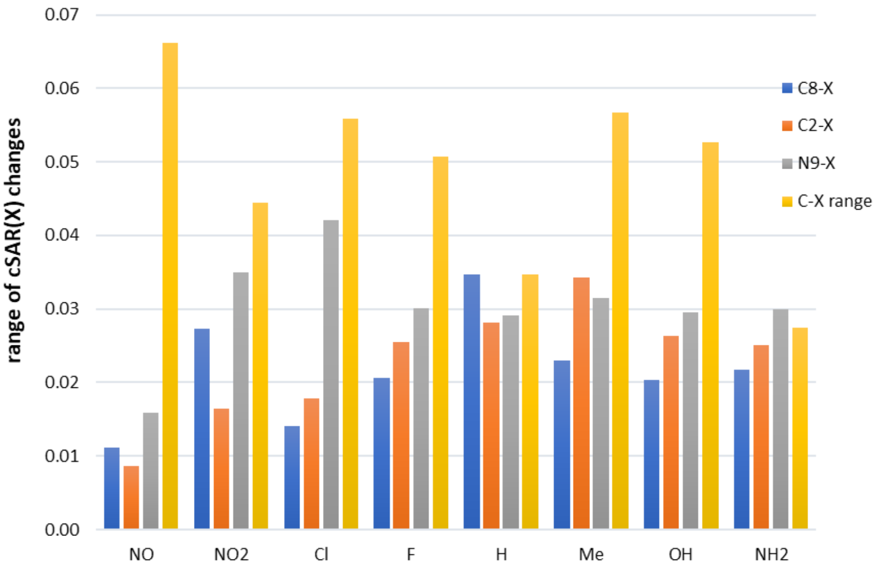

Figure S9. Ranges of cSAR(X) changes for the examined X substituent attached to C2, C8, and the nitrogen (N9) and carbon atoms in the adenine monomer and its pairs (WC, HG, and adenine-adenine).

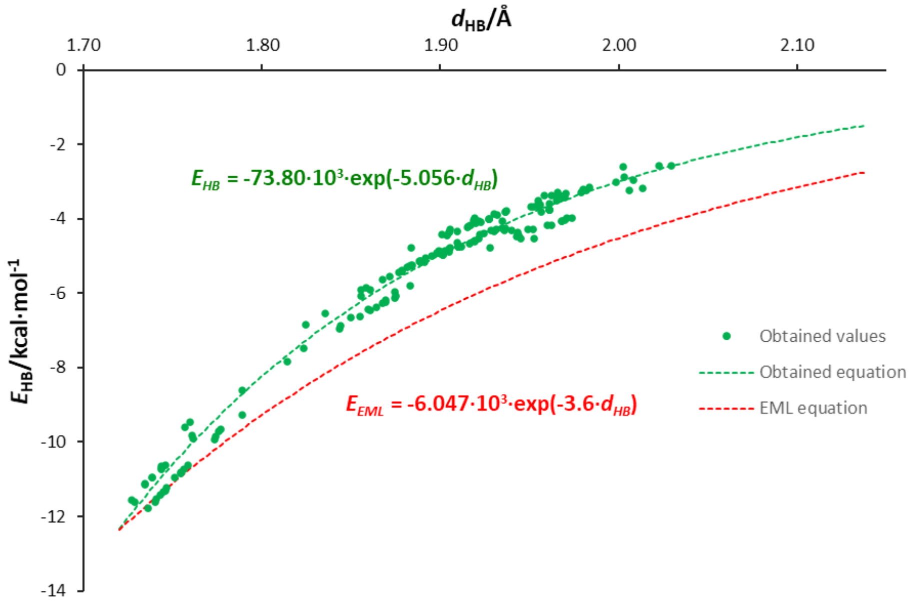

Figure S10. Dependence of H-bond energies, E_{HB}, on their lengths, d_{HB}, for all hydrogen bonds in studied adenine-uracil and adenine-adenine base pairs.

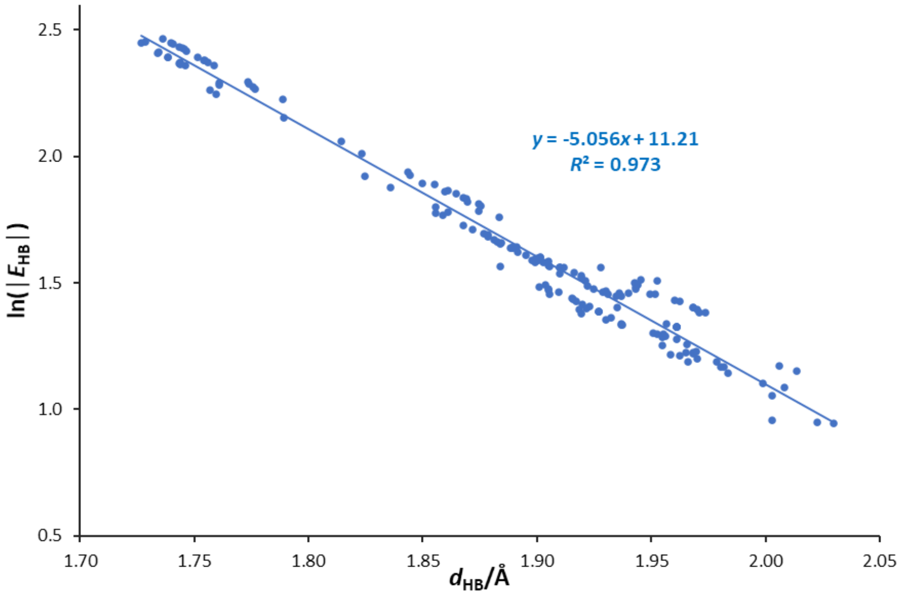

Figure S11. Relationship between ln(E_{HB}) and H-bond lengths, d_{HB}, for all hydrogen bonds in studied adenine-uracil and adenine-adenine base pairs.

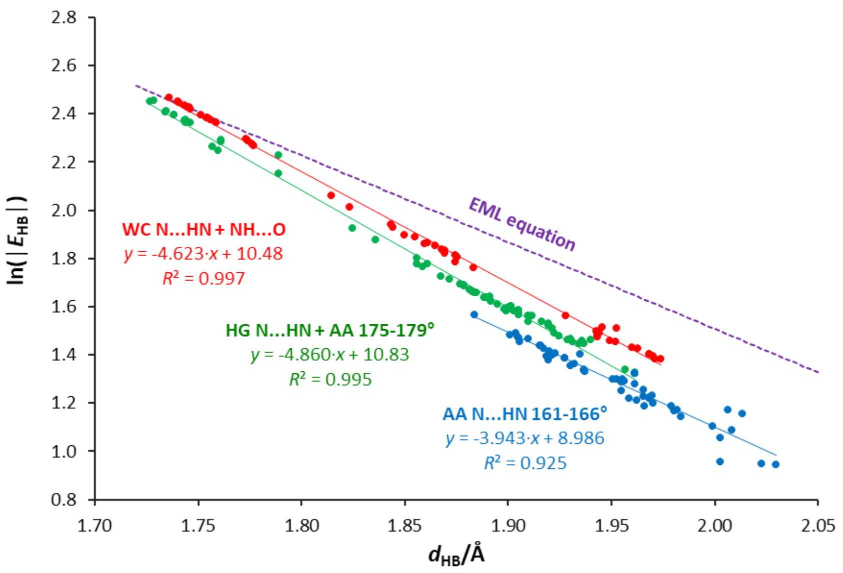

Figure S12. Relationships between ln(|E_{HB}|) and hydrogen bond lengths, d_{HB}, for the groups shown in Figure 8.

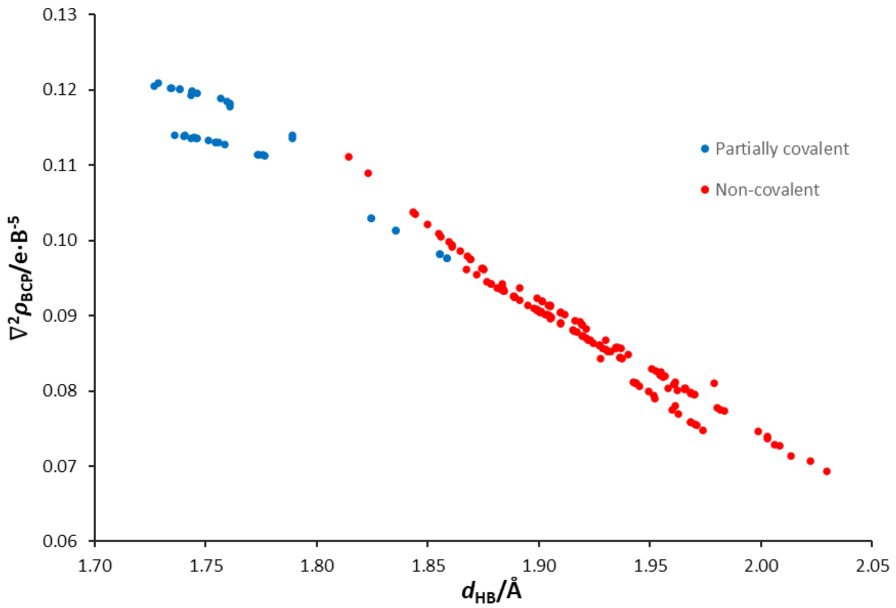

Figure S13. Dependence of Laplacian electron density, $\nabla^2\rho_{BCP}$, on the length of the H-bond, d_{HB}, for all hydrogen bonds in studied adenine-uracil and adenine-adenine base pairs.

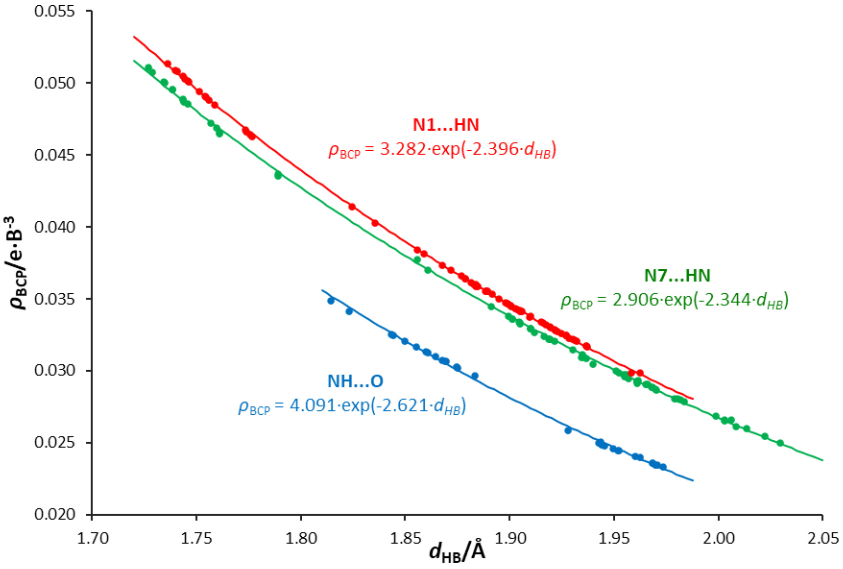

Figure S14. Dependences of electron density at the H-bond critical points, ρ_{BCP}, on their lengths, d_{HB}, in the studied adenine-uracil and adenine-adenine base pairs.

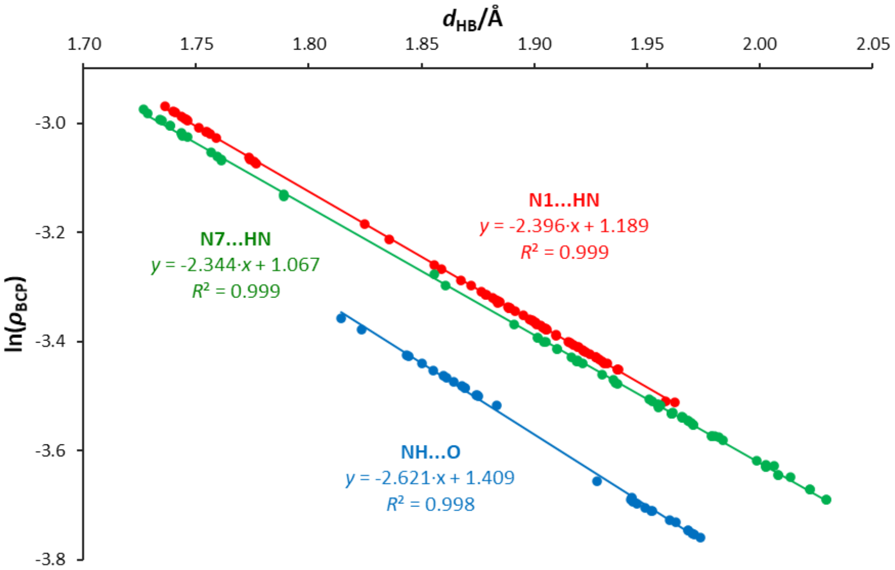

Figure S15. Relationships between ln(ρ_{BCP}) and H-bond lengths, d_{HB}, for all hydrogen bonds in studied adenine-uracil and adenine-adenine base pairs.

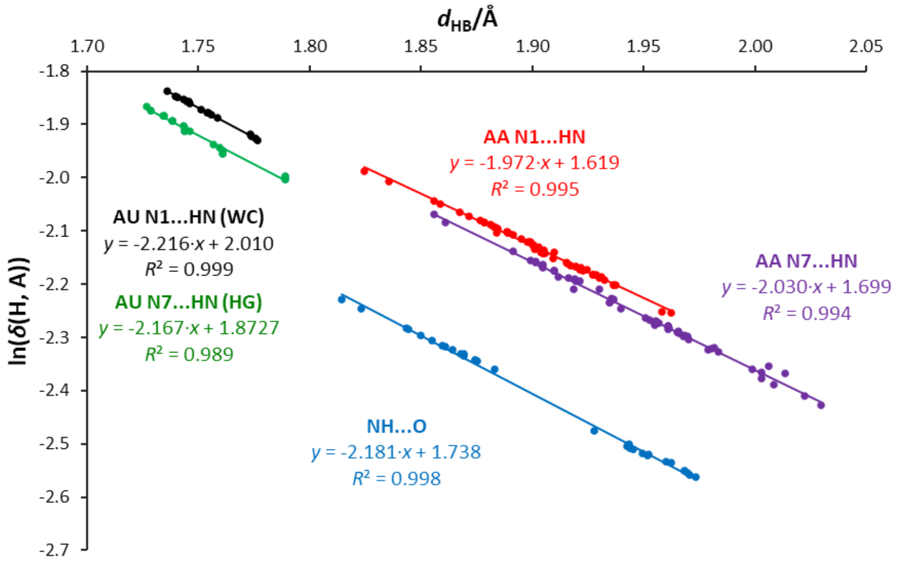

Figure S16. Relationships between ln(DI) and H-bond lengths, d_{HB}, for all hydrogen bonds in studied adenine-uracil and adenine-adenine base pairs.

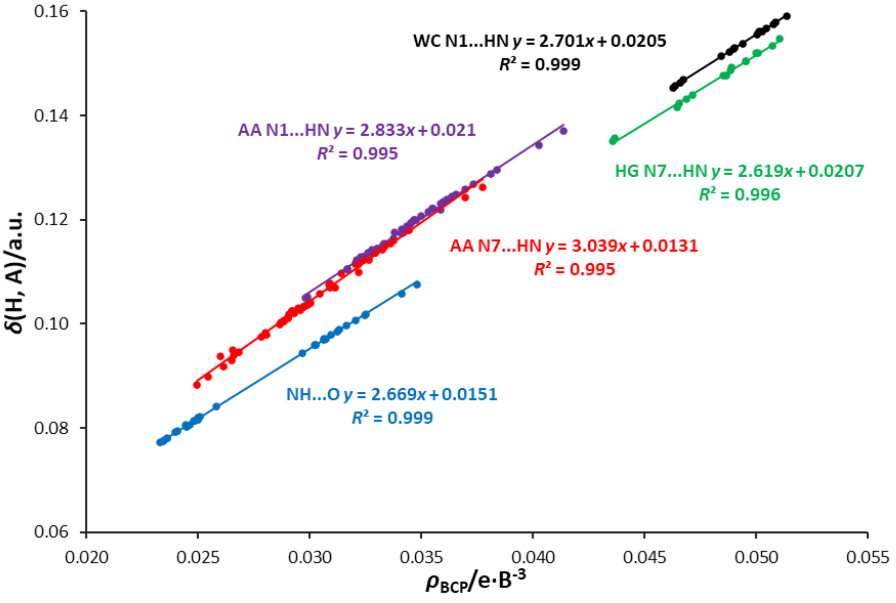

Figure S17. Delocalization index δ(H,A) between H and A atoms, where A is the acceptor of H-bond as a function of electron density ρ_{BCP} at H-bond critical point for AU and AA dimers.

Table S1. Values of cSAR(X), cSAR(NH_2) and HOMA. Substituted adenine-uracil Hoogsteen (HG) and Watson-Crick (WC) pairs. HOMA values of five-membered adenine ring (HOMA AD5) and six-membered adenine ring (HOMA AD6).

HG C8-X	X=	*c*SAR(X)	*c*SAR(NH_2)	HOMA AD5	HOMA AD6
	NO	-0.063	0.125	0.807	0.828
	NO_2	-0.055	0.117	0.815	0.863
	Cl	0.110	0.088	0.781	0.910
	F	0.126	0.088	0.758	0.920
	H	0.132	0.097	0.816	0.918
	CH_3	0.133	0.077	0.788	0.911
	OH	0.100	0.079	0.780	0.936
	NH_2	0.185	0.069	0.771	0.933
	Range	***0.248***	***0.056***	***0.057***	***0.108***
**HG C2-X**	NO	-0.033	0.224	0.829	0.899
	NO_2	-0.036	0.234	0.828	0.911
	Cl	0.044	0.219	0.823	0.923
	F	0.072	0.216	0.821	0.920
	H	0.105	0.198	0.816	0.918
	CH_3	0.119	0.193	0.817	0.919
	OH	0.141	0.200	0.814	0.922
	NH_2	0.206	0.189	0.807	0.912
	Range	***0.262***	***0.045***	***0.022***	***0.024***
**HG N9-X**	NO	-0.017	0.212	0.693	0.924
	NO_2	-0.023	0.214	0.700	0.918
	Cl	0.055	0.206	0.794	0.916
	F	0.041	0.210	0.823	0.909
	H	0.132	0.198	0.816	0.918
	CH_3	0.137	0.195	0.809	0.920
	OH	0.098	0.207	0.861	0.914
	NH_2	0.129	0.198	0.820	0.919
	Range	***0.160***	***0.019***	***0.167***	***0.015***
**WC C8-X**	NO	-0.087	0.253	0.806	0.836
	NO_2	-0.062	0.242	0.825	0.875
	Cl	0.085	0.211	0.790	0.920
	F	0.101	0.206	0.767	0.927
	H	0.115	0.207	0.809	0.916
	CH_3	0.154	0.199	0.799	0.922
	OH	0.173	0.190	0.782	0.931
	NH_2	0.211	0.187	0.781	0.932
	Range	***0.299***	***0.066***	***0.058***	***0.096***
**WC N9-X**	NO	-0.031	0.221	0.924	0.927
	NO_2	-0.035	0.222	0.918	0.921
	Cl	0.045	0.215	0.916	0.916
	F	0.032	0.217	0.909	0.909
	H	0.103	0.210	0.918	0.916
	CH_3	0.120	0.205	0.920	0.917
	OH	0.090	0.216	0.914	0.912
	NH_2	0.121	0.209	0.919	0.919
	Range	***0.156***	***0.018***	***0.015***	***0.019***

Table S2. Values of cSAR(X), cSAR(NH_2) and HOMA. Substituted adenine-adenine AA2 and AA3 pairs. HOMA values of five-membered adenine ring (HOMA AD5) and six-membered adenine ring (HOMA AD6).

**AA2 C2-X**	**X**	***c*SAR(X)**	***c*SAR(NH_2)**	**HOMA AD5**	**HOMA AD6**
	NO	-0.024	0.193	0.807	0.890
	NO_2	-0.036	0.207	0.819	0.894
	Cl	0.043	0.193	0.814	0.908
	F	0.071	0.193	0.812	0.905
	H	0.104	0.174	0.807	0.905
	CH_3	0.125	0.169	0.808	0.906
	OH	0.141	0.176	0.805	0.909
	NH_2	0.205	0.166	0.799	0.899
	Range	***0.241***	***0.041***	***0.020***	***0.019***
**AA2 C8-X**	NO	-0.080	0.221	0.812	0.837
	NO_2	-0.063	0.214	0.829	0.874
	Cl	0.086	0.184	0.792	0.920
	F	0.101	0.179	0.769	0.926
	H	0.104	0.174	0.810	0.913
	CH_3	0.154	0.173	0.800	0.922
	OH	0.173	0.165	0.783	0.931
	NH_2	0.212	0.160	0.782	0.932
	Range	***0.292***	***0.061***	***0.060***	***0.095***
**AA2 N9-X**	NO	-0.028	0.189	0.683	0.911
	NO_2	-0.031	0.190	0.689	0.905
	Cl	0.050	0.182	0.786	0.902
	F	0.036	0.185	0.812	0.893
	H	0.107	0.174	0.807	0.905
	CH_3	0.131	0.172	0.811	0.905
	OH	0.092	0.183	0.853	0.900
	NH_2	0.124	0.175	0.802	0.907
	Range	***0.163***	***0.019***	***0.170***	***0.018***
**AA3 C2-X**	NO	-0.024	0.193	0.809	0.896
	NO_2	-0.020	0.208	0.817	0.900
	Cl	0.045	0.193	0.814	0.914
	F	0.072	0.193	0.812	0.911
	H	0.105	0.170	0.807	0.912
	CH_3	0.126	0.170	0.812	0.911
	OH	0.141	0.176	0.806	0.914
	NH_2	0.206	0.161	0.800	0.900
	Range	***0.231***	***0.047***	***0.017***	***0.018***
**AA3 N9-X**	NO	-0.026	0.189	0.686	0.917
	NO_2	-0.030	0.190	0.690	0.911
	Cl	0.051	0.182	0.786	0.908
	F	0.037	0.185	0.813	0.899
	H	0.107	0.170	0.807	0.912
	CH_3	0.124	0.172	0.813	0.913
	OH	0.093	0.183	0.854	0.906
	NH_2	0.125	0.175	0.803	0.913
	Range	***0.155***	***0.020***	***0.168***	***0.018***

Table S3. Values of cSAR(X), cSAR(NH_2) and HOMA. Substituted adenine-adenine AA4 pairs. HOMA values of five-membered adenine ring (HOMA AD5) and six-membered adenine ring (HOMA AD6).

**AA4 C8-X**	**X**	***c*SAR(X)**	***c*SAR(NH_2)**	**HOMA AD5**	**HOMA AD6**
	NO	-0.090	0.226	0.810	0.832
	NO_2	-0.064	0.197	0.829	0.870
	Cl	0.085	0.187	0.791	0.917
	F	0.101	0.183	0.769	0.924
	H	0.115	0.184	0.811	0.912
	CH_3	0.153	0.176	0.800	0.919
	OH	0.173	0.168	0.783	0.929
	NH_2	0.211	0.165	0.782	0.929
	Range	***0.301***	***0.062***	***0.060***	***0.097***
**AA4 N9-X**	NO	-0.032	0.196	0.677	0.923
	NO_2	-0.037	0.197	0.689	0.917
	Cl	0.043	0.190	0.790	0.912
	F	0.030	0.193	0.821	0.905
	H	0.102	0.184	0.811	0.912
	CH_3	0.127	0.182	0.815	0.912
	OH	0.089	0.192	0.856	0.908
	NH_2	0.120	0.185	0.806	0.915
	Range	***0.163***	***0.015***	***0.179***	***0.018***

Table S4. Values of cSAR(X) and cSAR(NH_2). Symmetrically substituted adenine-adenine AA3 and AA4 pairs.

**AA3 C2-X+C2-X**	**X**	***c*SAR(X)**	***c*SAR(NH_2)**
	NO	-0.018	0.191
	NO_2	-0.050	0.209
	Cl	0.047	0.194
	F	0.074	0.194
	H	0.105	0.170
	CH_3	0.125	0.168
	OH	0.142	0.176
	NH_2	0.203	0.159
	Range	***0.253***	***0.050***
**AA4 C8-X+C8-X**	NO	-0.069	0.228
	NO_2	-0.053	0.221
	Cl	0.087	0.188
	F	0.102	0.184
	H	0.115	0.184
	CH_3	0.152	0.175
	OH	0.172	0.167
	NH_2	0.208	0.163
	Range	***0.277***	***0.065***

Table S5. Values of cSAR(X) and cSAR(NH_2). Asymmetrically substituted adenine-adenine AA2 pairs.

AA2 C2-X+C8-X	X	*c*SAR(X)$_{C8X}$	*c*SAR(NH_2)$_{C8X}$	*c*SAR(NH_2)$_{C2X}$	*c*SAR(NH2)$_{C8X}$
	NO	-0.015	-0.072	0.196	0.223
	NO_2	-0.049	-0.053	0.210	0.217
	Cl	0.045	0.088	0.194	0.185
	F	0.072	0.103	0.194	0.181
	H	0.104	0.174	0.181	0.181
	CH_3	0.124	0.153	0.168	0.172
	OH	0.140	0.174	0.176	0.176
	NH_2	0.202	0.209	0.165	0.160
	Range	***0.252***	***0.281***	***0.045***	***0.064***

Table S6. HOMA values of uracil ring in adenine-uracil Hoogsteen (HG) and Watson-Crick (WC) pairs with substituents at C2, C8, N9 position of adenine moiety.

X	WC C8-X	HG C2-X	WC N9-X
NO	0.572	0.530	0.565
NO_2	0.570	0.530	0.565
Cl	0.559	0.526	0.561
F	0.558	0.526	0.562
H	0.556	0.524	0.556
CH_3	0.554	0.523	0.555
OH	0.555	0.523	0.560
NH_2	0.552	0.523	0.557
Range	***0.020***	***0.007***	***0.011***

Table S7. Calculated hydrogen bond parameters of all studied base pairs. Energy of individual hydrogen bond obtained with NBO approach E_{HB}/kcal·mol^{-1}; Interaction energy obtained with supermolecular method E_{SM}/kcal·mol^{-1}; total deformation energy E_{def}/kcal·mol^{-1}; hydrogen bond length d/Å; electron density at hydrogen bond critical point ρ_{BCP}/e·B^{-3}; Laplacian of electron density at hydrogen bond critical point L_{BCP}/ e·B^{-5}; delocalization index between hydrogen atom and hydrogen bond acceptor atom δ(H, A)/a.u.

		HB1					HB2							
	X	***d***	**ρ_{BCP}·10^2**	**L_{BCP}·10^2**	**δ(H, A)·10^2**	**E_{HB}**	***d***	**ρ_{BCP}·10^2**	**L_{BCP}·10^2**	**δ(H, A)·10^2**	**E_{HB}**	**∑E_{HB}**	**E_{def}**	**E_{SM}**
**Hoogsteen C2-X**	NO	1.952	2.447	7.897	8.052	-4.52	1.760	4.689	11.85	14.32	-9.45	-13.97	1.48	-15.50
	NO_2	1.928	2.585	8.437	8.410	-4.77	1.757	4.718	11.89	14.40	-9.60	-14.37	0.70	-16.67
	Cl	1.945	2.481	8.056	8.129	-4.54	1.746	4.854	11.96	14.77	-10.61	-15.15	1.63	-15.78
	F	1.943	2.498	8.121	8.167	-4.48	1.744	4.883	11.98	14.86	-10.73	-15.21	1.67	-15.82
	H	1.968	2.361	7.587	7.801	-4.07	1.738	4.955	12.01	15.05	-10.95	-15.02	1.70	-15.61
	CH_3	1.974	2.330	7.483	7.717	-3.99	1.734	5.009	12.03	15.20	-11.11	-15.1	1.76	-15.60
	OH	1.952	2.449	7.933	8.029	-4.28	1.735	5.002	12.02	15.19	-11.15	-15.43	1.67	-15.98
	NH_2	1.971	2.346	7.548	7.745	-3.98	1.727	5.107	12.05	15.47	-11.57	-15.55	1.92	-15.69

		HB1					HB2							
	X	d	$\rho_{BCP}\cdot10^2$	$L_{BCP}\cdot10^2$	$\delta(H, A)\cdot10^2$	E_{HB}	d	$\rho_{BCP}\cdot10^2$	$L_{BCP}\cdot10^2$	$\delta(H, A)\cdot10^2$	E_{HB}	$\sum E_{HB}$	E_{def}	E_{SM}
**Hoogsteen N9-X**	NO	1.943	2.502	8.110	8.191	-4.37	1.789	4.369	11.36	13.57	-9.27	-13.64	1.34	-14.54
	NO_2	1.943	2.508	8.105	8.210	-4.41	1.789	4.356	11.39	13.50	-8.61	-13.02	1.36	-14.53
	Cl	1.960	2.408	7.751	7.937	-4.18	1.761	4.656	11.78	14.23	-9.90	-14.08	1.44	-14.99
	F	1.963	2.398	7.688	7.922	-4.16	1.761	4.649	11.82	14.17	-9.81	-13.97	1.43	-15.04
	H	1.968	2.361	7.587	7.801	-4.07	1.738	4.955	12.01	15.05	-10.95	-15.02	1.70	-15.61
	CH_3	1.970	2.349	7.554	7.766	-4.03	1.729	5.074	12.10	15.34	-11.62	-15.65	1.83	-15.81
	OH	1.944	2.488	8.099	8.143	-4.44	1.744	4.868	11.99	14.77	-10.64	-15.08	1.66	-15.49
	NH_2	1.949	2.462	7.995	8.064	-4.29	1.743	4.890	11.926	14.923	-10.69	-14.98	1.74	-15.38

		HB1					HB2							
	X	d	$\rho_{BCP}\cdot10^2$	$L_{BCP}\cdot10^2$	$\delta(H, A)\cdot10^2$	E_{HB}	d	$\rho_{BCP}\cdot10^2$	$L_{BCP}\cdot10^2$	$\delta(H, A)\cdot10^2$	E_{HB}	$\sum E_{HB}$	E_{def}	E_{SM}
**Watson-Crick C8-X**	NO	1.814	3.484	11.11	10.76	-7.85	1.777	4.627	11.13	14.53	-9.65	-17.5	1.78	-16.07
	NO_2	1.823	3.412	10.89	10.58	-7.48	1.776	4.640	11.13	14.58	-9.72	-17.20	1.76	-15.92
	Cl	1.861	3.124	9.946	9.85	-6.46	1.755	4.899	11.30	15.29	-10.83	-17.29	1.91	-15.64
	F	1.864	3.099	9.860	9.788	-6.37	1.754	4.904	11.30	15.29	-10.82	-17.19	1.91	-15.60
	H	1.868	3.075	9.786	9.722	-6.27	1.745	5.026	11.37	15.62	-11.34	-17.61	2.03	-15.68
	CH_3	1.875	3.023	9.611	9.589	-6.07	1.740	5.087	11.39	15.79	-11.60	-17.67	2.09	-15.65
	OH	1.875	3.028	9.627	9.604	-5.95	1.743	5.044	11.36	15.67	-11.41	-17.36	2.11	-15.55
	NH_2	1.883	2.967	9.423	9.442	-5.81	1.736	5.137	11.40	15.92	-11.79	-17.6	2.22	-15.51

		HB1					HB2							
	X	***d***	$\rho_{BCP}\cdot10^2$	$L_{BCP}\cdot10^2$	$\delta(H, A)\cdot10^2$	E_{HB}	***d***	$\rho_{BCP}\cdot10^2$	$L_{BCP}\cdot10^2$	$\delta(H, A)\cdot10^2$	E_{HB}	$\sum E_{HB}$	E_{def}	E_{SM}
**Watson-Crick N9-X**	NO	1.844	3.247	10.35	10.17	-6.87	1.773	4.674	11.14	14.69	-9.93	-16.8	1.74	-15.60
	NO_2	1.843	3.254	10.37	10.19	-6.96	1.774	4.663	11.14	14.64	-9.85	-16.81	1.74	-15.59
	Cl	1.855	3.167	10.09	9.965	-6.61	1.756	4.881	11.30	15.23	-10.74	-17.35	1.90	-15.71
	F	1.850	3.205	10.22	10.06	-6.65	1.759	4.846	11.28	15.14	-10.61	-17.26	1.88	-15.73
	H	1.869	3.065	9.746	9.722	-6.25	1.746	5.013	11.36	15.62	-11.31	-17.56	2.00	-15.68
	CH_3	1.874	3.028	9.624	9.601	-6.12	1.741	5.078	11.40	15.75	-11.54	-17.66	2.11	-15.66
	OH	1.860	3.134	9.976	9.881	-6.43	1.751	4.941	11.34	15.38	-10.96	-17.39	1.95	-15.78
	NH_2	1.869	3.063	9.744	9.692	-6.17	1.746	5.005	11.36	15.56	-11.23	-17.4	1.98	-15.71

		HB1					HB2							
	X	***d***	$\rho_{BCP}\cdot10^2$	$L_{BCP}\cdot10^2$	$\delta(H, A)\cdot10^2$	E_{HB}	***d***	$\rho_{BCP}\cdot10^2$	$L_{BCP}\cdot10^2$	$\delta(H, A)\cdot10^2$	E_{HB}	$\sum E_{HB}$	E_{def}	E_{SM}
**AA2 C2-X**	NO	1.901	3.446	9.063	11.83	-4.41	1.937	3.087	8.562	10.76	-4.25	-8.66	1.48	-13.10
	NO_2	1.884	3.586	9.358	12.19	-4.78	1.936	3.093	8.584	10.78	-4.30	-9.08	1.22	-13.85
	Cl	1.904	3.423	9.014	11.77	-4.44	1.919	3.218	8.877	11.15	-4.61	-9.05	1.49	-13.48
	F	1.905	3.415	8.997	11.74	-4.37	1.916	3.242	8.930	11.22	-4.66	-9.03	1.48	-13.54
	H	1.927	3.244	8.605	11.28	-4.00	1.910	3.294	9.039	11.37	-4.76	-8.76	1.43	-13.21
	CH_3	1.932	3.206	8.525	11.17	-3.90	1.905	3.334	9.125	11.49	-4.88	-8.78	1.45	-13.22
	OH	1.917	3.319	8.787	11.47	-4.16	1.901	3.361	9.196	11.56	-4.96	-9.12	1.41	-13.73
	NH_2	1.937	3.169	8.436	11.06	-3.79	1.891	3.445	9.376	11.80	-5.17	-8.96	1.55	-13.38

		HB1					HB2							
	X	*d*	$\rho_{BCP}\cdot10^2$	$L_{BCP}\cdot10^2$	$\delta(H, A)\cdot10^2$	E_{HB}	*d*	$\rho_{BCP}\cdot10^2$	$L_{BCP}\cdot10^2$	$\delta(H, A)\cdot10^2$	E_{HB}	$\sum E_{HB}$	E_{def}	E_{SM}
**AA2 C8-X**	NO	1.856	3.775	10.05	12.63	-6.06	1.958	2.989	8.036	10.52	-3.38	-9.44	1.49	-13.61
	NO_2	1.861	3.700	9.917	12.43	-5.92	1.962	2.984	8.009	10.51	-3.36	-9.28	1.44	-13.45
	Cl	1.901	3.361	9.194	11.55	-4.96	1.937	3.170	8.443	11.05	-3.81	-8.77	1.41	-13.15
	F	1.904	3.337	9.140	11.48	-4.86	1.937	3.171	8.443	11.06	-3.80	-8.66	1.42	-13.08
	H	1.910	3.294	9.039	11.37	-4.76	1.927	3.244	8.605	11.28	-4.00	-8.76	1.43	-13.21
	CH_3	1.919	3.222	8.873	11.18	-4.58	1.920	3.302	8.736	11.44	-4.12	-8.70	1.46	-13.18
	OH	1.921	3.208	8.829	11.15	-4.52	1.923	3.276	8.680	11.36	-4.08	-8.60	1.51	-13.01
	NH_2	1.930	3.143	8.677	10.97	-4.34	1.915	3.337	8.810	11.54	-4.22	-8.56	1.53	-13.02

		HB1					HB2							
	X	*d*	$\rho_{BCP}\cdot10^2$	$L_{BCP}\cdot10^2$	$\delta(H, A)\cdot10^2$	E_{HB}	*d*	$\rho_{BCP}\cdot10^2$	$L_{BCP}\cdot10^2$	$\delta(H, A)\cdot10^2$	E_{HB}	$\sum E_{HB}$	E_{def}	E_{SM}
**AA2 N9-X**	NO	1.906	3.412	8.970	11.75	-4.28	1.957	2.949	8.200	10.31	-3.81	-8.09	1.35	-12.90
	NO_2	1.905	3.419	8.987	11.75	-4.35	1.961	2.911	8.114	10.18	-3.77	-8.12	1.36	-12.78
	Cl	1.919	3.305	8.736	11.45	-3.97	1.935	3.091	8.578	10.70	-4.25	-8.22	1.35	-12.86
	F	1.916	3.332	8.797	11.52	-4.21	1.940	3.048	8.481	10.58	-4.31	-8.52	1.33	-12.82
	H	1.927	3.244	8.605	11.28	-4.00	1.910	3.294	9.039	11.37	-4.76	-8.76	1.43	-13.21
	CH_3	1.930	3.222	8.555	11.22	-3.88	1.899	3.377	9.238	11.60	-4.88	-8.76	1.50	-13.35
	OH	1.910	3.376	8.912	11.64	-4.33	1.912	3.265	9.019	11.22	-4.76	-9.09	1.48	-13.55
	NH_2	1.921	3.287	8.705	11.39	-4.05	1.905	3.328	9.140	11.43	-4.81	-8.86	1.49	-13.47

		HB1					HB2							
	X	***d***	**ρ_{BCP}·10^2**	**L_{BCP}·10^2**	**δ(H, A)·10^2**	**E_{HB}**	***d***	**ρ_{BCP}·10^2**	**L_{BCP}·10^2**	**δ(H, A)·10^2**	**E_{HB}**	**$\sum E_{HB}$**	**E_{def}**	**E_{SM}**
	NO	1.951	3.003	8.287	10.41	-3.67	2.003	2.662	7.394	9.390	-2.6	-6.27	1.84	-10.86
	NO_2	1.935	3.114	8.568	10.70	-4.06	1.999	2.685	7.466	9.453	-3.01	-7.07	1.20	-11.94
	Cl	1.953	2.989	8.263	10.36	-3.66	1.983	2.784	7.731	9.757	-3.14	-6.80	1.29	-11.76
**AA3 C2-X**	F	1.955	2.975	8.218	10.32	-3.62	1.980	2.804	7.782	9.819	-3.21	-6.83	1.28	-11.82
	H	1.961	2.924	8.084	10.24	-3.76	1.961	2.924	8.084	10.24	-3.76	-7.52	1.36	-11.84
	CH_3	2.014	2.602	7.139	9.377	-3.17	2.006	2.658	7.283	9.499	-3.23	-6.40	1.25	-11.58
	OH	1.968	2.885	7.983	10.06	-3.39	1.965	2.904	8.041	10.12	-3.41	-6.80	1.20	-11.95
	NH_2	1.955	2.959	8.255	10.27	-3.50	1.919	3.219	8.925	10.98	-4.03	-7.53	1.44	-12.01

		HB1					HB2							
	X	***d***	**ρ_{BCP}·10^2**	**L_{BCP}·10^2**	**δ(H, A)·10^2**	**E_{HB}**	***d***	**ρ_{BCP}·10^2**	**L_{BCP}·10^2**	**δ(H, A)·10^2**	**E_{HB}**	**$\sum E_{HB}$**	**E_{def}**	**E_{SM}**
	NO	1.955	2.975	8.199	10.34	-3.66	2.022	2.544	7.066	8.979	-2.58	-6.24	1.15	-11.16
	NO_2	1.956	2.971	8.185	10.31	-3.63	2.030	2.496	6.936	8.823	-2.57	-6.20	1.16	-11.03
	Cl	1.968	2.887	7.974	10.07	-3.40	2.003	2.651	7.368	9.289	-2.87	-6.27	1.17	-11.17
**AA3 N9-X**	F	1.965	2.906	8.025	10.13	-3.51	2.008	2.614	7.277	9.178	-2.97	-6.48	1.14	-11.11
	H	1.961	2.924	8.084	10.24	-3.76	1.961	2.924	8.084	10.24	-3.76	-7.52	1.36	-11.84
	CH_3	1.982	2.802	7.745	9.828	-3.22	1.966	2.905	8.030	10.11	-3.28	-6.50	1.34	-11.66
	OH	1.961	2.930	7.802	10.21	-3.59	1.979	2.807	8.097	9.792	-3.28	-6.87	1.28	-11.79
	NH_2	1.970	2.874	7.952	10.04	-3.42	1.970	2.868	7.951	9.987	-3.32	-6.74	1.29	-11.78

		HB1					HB2							
	X	***d***	$\rho_{BCP}\cdot10^2$	$L_{BCP}\cdot10^2$	$\delta(H, A)\cdot10^2$	E_{HB}	***d***	$\rho_{BCP}\cdot10^2$	$L_{BCP}\cdot10^2$	$\delta(H, A)\cdot10^2$	E_{HB}	$\sum E_{HB}$	E_{def}	E_{SM}
	NO	1.825	4.138	10.30	13.70	-6.84	1.931	3.210	8.524	11.23	-4.28	-11.12	1.58	-14.68
	NO_2	1.836	4.029	10.12	13.44	-6.54	1.929	3.229	8.568	11.28	-4.32	-10.86	1.52	-14.49
	Cl	1.879	3.638	9.426	12.43	-5.42	1.900	3.456	9.053	11.93	-4.95	-10.37	1.45	-14.11
**AA4 C8-X**	F	1.883	3.603	9.354	12.34	-5.27	1.901	3.454	9.047	11.93	-4.90	-10.17	1.44	-14.04
	H	1.889	3.550	9.244	12.20	-5.14	1.889	3.551	9.247	12.21	-5.15	-10.29	1.46	-14.14
	CH_3	1.899	3.469	9.077	11.99	-4.92	1.881	3.616	9.376	12.38	-5.31	-10.23	1.49	-14.10
	O_H	1.899	3.468	9.075	11.99	-4.87	1.885	3.589	9.323	12.30	-5.25	-10.12	1.53	-13.99
	NH_2	1.910	3.382	8.889	11.76	-4.65	1.877	3.658	9.453	12.50	-5.44	-10.09	1.56	-13.94

		HB1					HB2							
	X	***d***	$\rho_{BCP}\cdot10^2$	$L_{BCP}\cdot10^2$	$\delta(H, A)\cdot10^2$	E_{HB}	***d***	$\rho_{BCP}\cdot10^2$	$L_{BCP}\cdot10^2$	$\delta(H, A)\cdot10^2$	E_{HB}	$\sum E_{HB}$	E_{def}	E_{SM}
	NO	1.859	3.813	9.757	12.89	-5.85	1.922	3.281	8.679	11.43	-4.43	-10.28	1.44	-14.18
	NO_2	1.856	3.840	9.806	12.96	-5.91	1.925	3.261	8.636	11.38	-4.38	-10.29	1.46	-14.20
	Cl	1.872	3.697	9.542	12.59	-5.54	1.903	3.435	9.011	11.88	-4.86	-10.40	1.48	-14.21
**AA4 N9-X**	F	1.868	3.734	9.613	12.69	-5.62	1.905	3.414	8.968	11.82	-4.78	-10.40	1.47	-14.26
	H	1.889	3.550	9.244	12.20	-5.14	1.889	3.551	9.247	12.21	-5.15	-10.29	1.46	-14.14
	CH_3	1.895	3.501	9.143	12.07	-5.00	1.884	3.597	9.335	12.33	-5.24	-10.24	1.50	-14.09
	OH	1.879	3.639	9.424	12.44	-5.39	1.898	3.476	9.095	11.99	-4.91	-10.30	1.47	-14.10
	NH_2	1.888	3.555	9.256	12.22	-5.14	1.892	3.531	9.202	12.15	-5.06	-10.20	1.47	-14.06

		HB1				HB2						
	X	***d***	ρ_{BCP}*10^2	L_{BCP}*10^2	E_{HB}	***d***	ρ_{BCP}*10^2	L_{BCP}*10^2	E_{HB}	$\sum E_{HB}$	E_{def}	E_{SM}
**AA2 C2-X+C8-X**	NO	1.886	3.482	9.481	-5.30	1.936	3.171	8.469	-3.77	-9.07	1.99	-12.36
	NO2	1.894	3.413	9.337	-5.13	1.925	3.247	8.645	-4.02	-9.15	1.39	-12.98
	Cl	1.911	3.343	9.019	-4.77	1.914	3.279	8.850	-4.26	-9.03	1.47	-13.34
	F	1.911	3.328	9.034	-4.75	1.916	3.285	8.809	-4.14	-8.89	1.45	-13.37
	H	1.910	3.294	9.039	-4.76	1.927	3.244	8.605	-4.00	-8.76	1.43	-13.21
	CH3	1.913	3.269	8.981	-4.68	1.927	3.249	8.617	-3.98	-8.66	1.48	-13.16
	OH	1.911	3.289	9.034	-4.70	1.916	3.331	8.802	-4.18	-8.88	1.47	-13.56
	NH2	1.912	3.247	8.608	-4.69	1.927	3.284	9.008	-3.96	-8.65	1.63	-13.09

		HB1				HB2						
	X	***d***	ρ_{BCP}*10^2	L_{BCP}*10^2	E_{HB}	***d***	ρ_{BCP}*10^2	L_{BCP}*10^2	E_{HB}	$\sum E_{HB}$	E_{def}	E_{SM}
**AA3 C2-X+C2-X**	NO	1.964	2.896	8.034	-3.67	1.964	2.895	8.032	-3.67	-7.34	2.58	-10.12
	NO2	1.968	2.877	7.986	-3.07	1.969	2.882	8.004	-3.07	-6.14	1.25	-11.29
	Cl	1.962	2.918	8.091	-3.62	1.963	2.923	8.107	-3.61	-7.23	1.33	-11.86
	F	1.961	2.922	8.095	-3.48	1.962	2.929	8.114	-3.48	-6.96	1.30	-12.00
	H	1.961	2.924	8.084	-3.76	1.961	2.924	8.084	-3.76	-7.52	1.36	-11.84
	CH3	1.975	2.846	7.876	-3.28	1.975	2.844	7.870	-3.28	-6.56	1.32	-11.56
	OH	1.959	2.945	8.150	-3.47	1.961	2.937	8.124	-3.46	-6.93	1.15	-12.37
	NH2	1.953	2.983	8.234	-3.84	1.954	2.977	8.215	-3.84	-7.68	1.49	-12.09

		HB1				HB2						
	X	***d***	ρ_{BCP}***10**^{2}	L_{BCP}***10**^{2}	E_{HB}	***d***	ρ_{BCP}***10**^{2}	L_{BCP}***10**^{2}	E_{HB}	$\sum E_{HB}$	E_{def}	E_{SM}
**AA4 C8-X+C8-X**	NO	1.872	3.686	9.534	-5.53	1.872	3.687	9.537	-5.53	-11.06	1.48	-14.17
	NO2	1.879	3.619	9.401	-5.36	1.880	3.624	9.415	-5.37	-10.73	1.40	-13.98
	Cl	1.890	3.535	9.221	-5.14	1.891	3.540	9.234	-5.15	-10.29	1.42	-14.03
	F	1.893	3.513	9.176	-5.04	1.893	3.517	9.186	-5.04	-10.08	1.43	-13.90
	H	1.889	3.551	9.247	-5.15	1.889	3.550	9.244	-5.14	-10.29	1.46	-14.14
	CH3	1.891	3.531	9.203	-5.07	1.892	3.531	9.203	-5.07	-10.14	1.50	-14.04
	OH	1.895	3.504	9.152	-4.97	1.895	3.503	9.148	-4.97	-9.94	1.59	-14.38
	NH2	1.897	3.488	9.111	-4.92	1.897	3.486	9.105	-4.92	-9.84	1.65	-13.65

Table S8. Changes in aromaticity expressed by the HOMA index for (a) AD6 and (b) AD5 rings due to substitution in WC and HG pairs.

(a)	**WC C8-X**	**HG C2-X**	**HG N9-X**	**WC N9-X**
**X**	Differences in HOMA index between substituted and unsubstituted systems*			
NO	0.079	0.019	-0.006	-0.011
NO_2	0.041	0.007	0.000	-0.005
Cl	-0.005	-0.005	0.002	-0.001
F	-0.011	-0.002	0.009	0.007
**H**	**0.000**	**0.000**	**0.000**	**0.000**
CH_3	-0.007	-0.001	-0.002	-0.001
OH	-0.016	-0.004	0.004	0.004
NH_2	-0.016	0.006	-0.001	-0.003
Differences in HOMA and cSAR(X) values for X=NH_2 and NO_2 systems**				
**ΔHOMA**	0.057	0.001	0.001	-0.002
**ΔcSAR(X)**	0.273	0.241	0.155	0.157
**ΔHOMA/ ΔcSAR(X)**	0.210	0.006	0.004	-0.015

(b)	**WC C8-X**	**HG C2-X**	**HG N9-X**	**WC N9-X**
**X**	Differences in HOMA index between substituted and unsubstituted systems*			
NO	0.003	-0.013	0.122	0.133
NO_2	-0.016	-0.012	0.116	0.121
Cl	0.019	-0.007	0.021	0.022
F	0.042	-0.005	-0.007	-0.009
**H**	**0.000**	**0.000**	**0.000**	**0.000**
CH_3	0.011	-0.001	0.006	-0.004
OH	0.027	0.002	-0.045	-0.045
NH_2	0.028	0.009	-0.004	0.005
Differences in HOMA and cSAR(X) values for X=NH_2 and NO_2 systems**				
**ΔHOMA**	-0.044	-0.021	0.120	0.116
**ΔcSAR(X)**	0.273	0.241	0.152	0.156
**ΔHOMA/ ΔcSAR(X)**	-0.163	-0.086	0.792	0.748

* HOMA(unsubstituted)-HOMA(substituted)

** ΔHOMA = HOMA(NH_2) - HOMA(NO_2); ΔcSAR(X) = cSAR(NH_2) - cSAR(NO_2).

Table S9. Changes in aromaticity expressed by the HOMA index for (a) AD6 and (b) AD5 rings due to substitution in AA dimers.

**(a)**	**AA2 C2-X**	**AA3 C2-X**	**AA2 C8-X**	**AA4 C8-X**	**AA2 N9-X**	**AA3 N9-X**	**AA4 N9-X**
**X**	Differences in HOMA index between substituted and unsubstituted systems*						
NO	0.027	0.016	0.071	0.083	0.007	-0.005	-0.008
NO_2	0.023	0.012	0.034	0.045	0.014	0.003	-0.002
Cl	0.008	-0.003	-0.014	-0.004	0.015	0.004	0.001
F	0.010	-0.001	-0.021	-0.010	0.024	0.013	0.009
**H**	**0.000**	**0.000**	**0.000**	**0.000**	**0.000**	**0.000**	**0.000**
CH_3	0.008	0.002	-0.018	-0.008	0.009	-0.002	0.000
OH	0.006	-0.005	-0.027	-0.017	0.015	0.003	0.005
NH_2	0.014	0.005	-0.028	-0.018	0.007	-0.004	-0.002
Differences in HOMA and cSAR(X) values for X=NH_2 and NO_2 systems**							
**ΔHOMA**	0.005	0.000	0.058	0.059	0.002	0.003	-0.002
**ΔcSAR(X)**	0.241	0.226	0.274	0.275	0.152	0.156	0.156
**ΔHOMA/ ΔcSAR(X)**	0.020	-0.001	0.210	0.214	0.016	0.016	-0.010

**(b)**	**AA2 C2-X**	**AA3 C2-X**	**AA2 C8-X**	**AA4 C8-X**	**AA2 N9-X**	**AA3 N9-X**	**AA4 N9-X**
**X**	Differences in HOMA index between substituted and unsubstituted systems*						
NO	0.005	-0.001	-0.003	0.003	0.129	0.122	0.135
NO_2	-0.006	-0.009	-0.021	-0.016	0.124	0.118	0.123
Cl	-0.002	-0.006	0.015	0.020	0.026	0.022	0.022
F	0.000	-0.005	0.037	0.042	-0.001	-0.005	-0.009
**H**	**0.000**	**0.000**	**0.000**	**0.000**	**0.000**	**0.000**	**0.000**
CH_3	0.003	0.001	0.006	0.010	0.000	-0.005	-0.004
OH	0.006	0.002	0.023	0.028	-0.041	-0.047	-0.044
NH_2	0.012	0.006	0.024	0.028	0.009	0.004	0.005
Differences in HOMA and cSAR(X) values for X=NH_2 and NO_2 systems**							
**ΔHOMA**	-0.020	-0.017	-0.047	-0.046	0.113	0.112	0.117
**ΔcSAR(X)**	0.241	0.226	0.274	0.275	0.156	0.155	0.157
**ΔHOMA/ ΔcSAR(X)**	-0.083	-0.076	-0.172	-0.169	0.729	0.725	0.745

* HOMA(unsubstituted)-HOMA(substituted)

** ΔHOMA = HOMA(NH_2) - HOMA(NO_2); ΔcSAR(X) = cSAR(NH_2) - cSAR(NO_2).
